# Towards full optimisation of automated double electron–electron resonance spectroscopy

**DOI:** 10.1039/d5cp03536h

**Published:** 2025-12-08

**Authors:** Hugo Karas, Sergei Kuzin, Stefan Stoll, Gunnar Jeschke

**Affiliations:** a Department of Chemistry and Applied Biosciences, ETH Zürich Vladimir-Prelog-Weg 2 Zürich 8093 Switzerland gjeschke@ethz.ch; b Department of Chemistry, University of Washington Seattle Washington USA

## Abstract

The double electron–electron resonance (DEER) experiment is widely applied for measuring distance distributions in biological systems and synthetic materials. Optimal manual setup requires substantial expertise and effort. In order to make DEER more accessible and to improve the reliability and reproducibility, we lay out a highly optimised and fully automatable protocol for nitroxide–nitroxide DEER spectroscopy, utilising the latest developments in pulse sequences and data processing. Additionally, we present autoDEER, a Python-based software that enables automatic DEER measurements on a wide range of spectrometers, both home-built and commercial. We show that autoDEER is able to perform a DEER measurement after sample insertion at the push of a button, including all the necessary setup experiments as well as inference of the distance distribution from the measured data. We apply this protocol to a range of samples of current interest in biology, illustrating that it is both robust and generally applicable.

## Abstract 2

A detailed protocol for automatically measuring DEER spectroscopy is shown as well as its application to homebuilt and commercial spectrometers.

## Graphical abstract

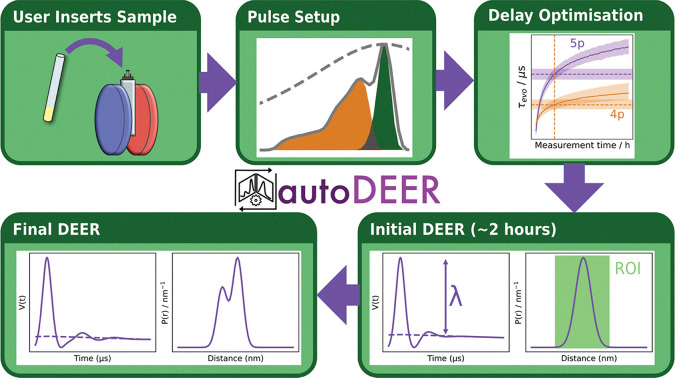

## 1 Introduction

In recent years, double electron–electron resonance (DEER) spectroscopy, also known as pulse electron–electron double resonance (PELDOR) spectroscopy has been increasingly used to study the structure and dynamics of biological macromolecules.^1–3^ DEER spectroscopy is a pulse electron paramagnetic resonance (EPR) technique^4^ that utilises the dipolar coupling between two spatially separated paramagnetic spin centres to determine the distribution of the distance between these spin centres within the sample.

In recent years, DEER experiments have been applied to increasingly more challenging samples. These samples often exhibit broad long-distance distributions or high local spin concentrations resulting in a low echo amplitude and requiring very long measurement times (>24 hours).^5–8^ In these cases it is critically important that the DEER experiment is optimally set up and that the most appropriate DEER pulse sequence is used. The inference of the distance distribution from the measured time-domain data is a mathematically ill-posed problem and requires rigorous data analysis. The interpretation of distance distributions for either ensemble structure modelling or identifying differences in structure upon binding or changes of protein environment require that the distance distribution is both reliable and reproducible. Recently, community standards for measurements and data analysis have been established for this purpose.^3^

**Fig. 1:**
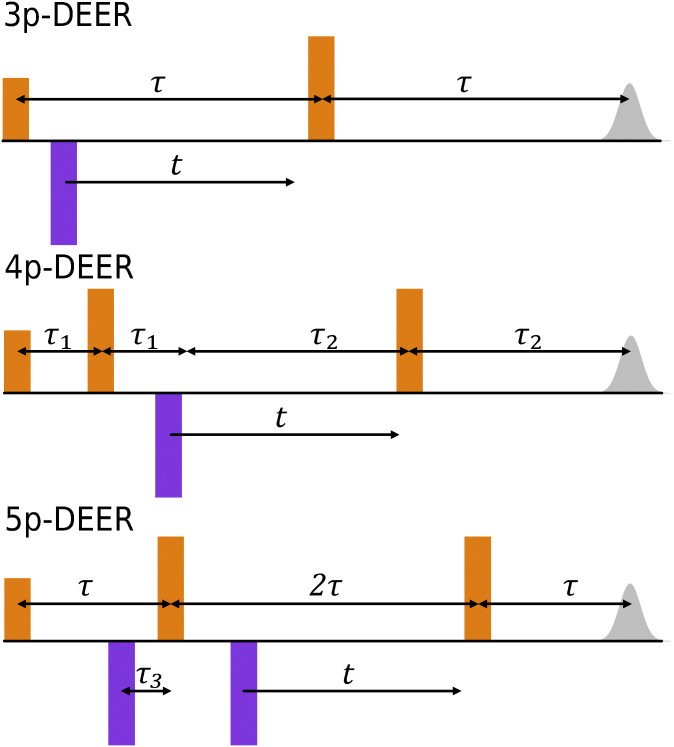
Comparison of the three-pulse, four-pulse and five-pulse DEER pulse sequences. The observer pulses are shown in orange, the pump pulses in purple and the detected echo in grey. The first pulse in each sequence is a π/2 and all subsequent pulses are π pulses.

DEER pulse sequences use two frequencies: an observer frequency by which a refocused spin echo is generated and observed, and a pump frequency. At least one time-varying inversion pulse is applied at this pump frequency to encode the distance information onto the observer echo in the form of an amplitude modulation. In the original three-pulse DEER experiment, which is still occasionally used, the observer sequence is a two-pulse Hahn echo.^1^ By extending the observer sequence to a refocused Hahn echo,^9,10^ the dead time for dipolar evolution is eliminated, allowing detection of broadly distributed distances at the low end of the accessible distance range. This four-pulse DEER experiment has since become the most widely applied DEER sequence, see Fig. 1.

The utility of DEER in biological applications depends on the distance range that can be accessed. The shortest distances are determined by the requirement that the excitation bandwidth of both observer and pump π pulses must exceed the dipole–dipole coupling.^11^ With high-power Q-band DEER, the shortest accessible distance is approximately 1.5 nm.^12^ The maximum distance that can be reliably extracted from a DEER experiment is dependent on the maximum sweep time of the pump pulse, the so called dipolar evolution time (*τ*_evo_). This time is predominately limited by loss of the electron spin coherence. For samples with a high proton concentration, many methyl groups^13,14^ or a comparatively high local electron spin concentration (such as proteins in a liquid droplet^15^), the decoherence can be very fast, preventing the data analysis for systems with long inter-spin distances. In recent years, a number of additional DEER pulse sequences have been developed with the aim of extending the electron coherence lifetime and thus allowing the measurement of longer distances.^16–18^ Many of these methods dynamically decouple the electron spin from the nuclear spin bath.

The most established of these techniques is the five-pulse DEER technique,^16^ see Fig. 1. An additional static pump pulse is added shortly before the first observer π pulse (delay *τ*_3_). This allows for observer inter-pulse delays *τ* and 2*τ* that meet the Carr–Purcell dynamical decoupling criterion. The maximum dipolar evolution time is close to 2*τ* − *τ*_3_. The dipolar coherence pathway that involves inversion by the static pump pulse is dominant and refocuses at *t* = *τ*_3_.^19–21^ The coherence pathway of four-pulse DEER typically also contributes, as some spin packets are not inverted by the static pump pulse.^16^ This pathway refocuses at *t* = *τ*. The signal contribution from this additional coherence pathway hindered the extraction of accurate distance distributions computed by established DEER data processing, which prevented five-pulse DEER becoming widely used. However, recently a multiple-pathway fitting approach has been implemented in the DEER data processing software DeerLab, which solves this problem.^21,22^ Although this experiment can be crucial in situations where coherence lifetime cannot be extended by deuteration of the solvent and protein,^15^ it is only slowly adopted by the community because of the increased effort for setup and data processing.

As DEER spectroscopy has become increasingly common, it has been recognised that recommendations for good practice are required to ensure reliability of distance distributions.^3^ These recommendations concern choice of measurement parameters, setup procedures, and data analysis. Clearly, the most robust and convenient way for meeting such standards is a fully optimised and automated protocol for performing DEER spectroscopy. Such automatisation is also required for DEER to be adopted beyond specialised EPR research labs and thus become accessible to the wider scientific community. This way non-EPR experts could apply DEER to their samples as an integrated approach with only basic knowledge of EPR spectroscopy and without detailed expertise on operating sophisticated EPR spectrometers.

As a point of departure for developing an optimised and automated protocol, we use the 2021 community-backed white paper on DEER spectroscopy.^3^ This white paper sets out key requirements for DEER spectroscopy and how to correctly process the data. Through benchmark measurements, this paper clearly demonstrated that DEER spectroscopy is a highly reproducible spectroscopic technique. However, many recent developments in DEER spectroscopy were excluded to keep the recommendations simple. In an optimised and automated protocol, the most important recent developments should be included to provide access to the full potential of DEER.

In this paper, we present a highly optimised protocol for DEER spectroscopy, based on the current state-of-the-art understanding. Implementation of this protocol is feasible on most modern spectrometers. We set out clear step-by-step instructions on how to optimise each parameter. In the cases of relaxation-limited samples the protocol optimises for the longest possible dipolar evolution time, with MNR (modulation-to-noise ratio) ≈ 150. For not relaxation-limited samples with ample signal, the shortest possible measurement time is optimised. We present a fully automated push-button implementation of the protocol, autoDEER, that can be run on a wide range of commercial and home-built spectrometers.

To validate the reliability and repeatability of the automated protocol, this protocol is validated on six test samples, using both a home-built and a commercial spectrometer. The test samples represent a wide spectrum of biologically relevant and interesting proteins, and have all been sourced from recently published work. The test set encompassed serine/arginine-rich splicing factor 1 (SRSF1),^23^ fused in sarcoma (FUS),^15^ polypyrimidine-tract binding protein 1 (PTBP1),^5^ Yersinia outer protein O (YopO),^3^ and maltodextrin binding protein (MBP).^24^ The samples have been prepared in a wide range of conditions, covering both sample and solvent protonation and deuteration. Notably, the YopO protein was widely measured and investigated as part of the community white paper.^3^ More information about the samples can be found in Section 4.1.

We present the complete protocol with detailed descriptions of each stage in Section 2, followed by an introduction to the autoDEER software package in Section 3. Section 4 describes the test samples and equipment used in this study. In Section 5, we demonstrate the performance of the protocol for all the test samples and compare them to measurements from literature. In this section we also demonstrate the protocol on commercial equipment. Finally, Section 6 discusses current limitations and future directions.

## 2 The protocol

In this section, we present an optimised protocol for conducting DEER experiments. While primarily designed for automation, this protocol can also be followed manually. Although developed for nitroxide–nitroxide DEER experiments, much of the protocol is broadly applicable and can be adapted for other spin labels or orthogonally spin-labelled samples.

**Fig. 2:**
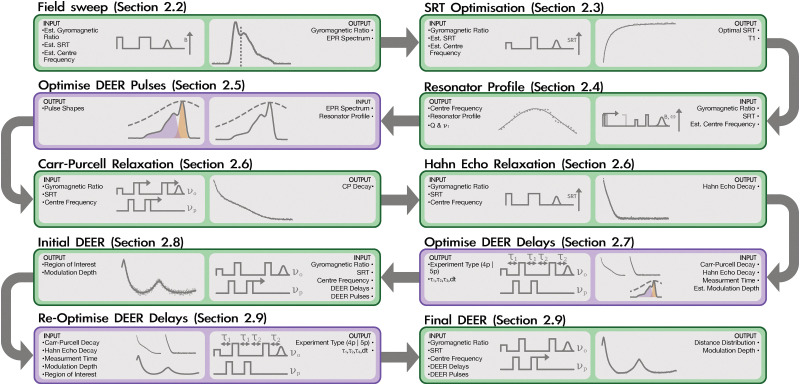
Flow chart of the DEER automation protocol. Green boxes represent the pulse experiments and their subsequent analysis, and purple boxes represent DEER-specific decisions. This protocol represents the default operating mode and shows the case where the five-pulse DEER sequence has been determined to be optimal. Not all feedback loops and steps are shown. The cases where the four-pulse sequence is optimal or the DEER sequence is fixed by the operator are analogous. Additional relaxation experiments might also be measured depending on the operating mode.

Fig. 2 provides an overview of the protocol in the form of a flow chart. The protocol consists of the following main steps:

(1) Sample insertion

(2) Field sweep

(3) Shot repetition time (SRT) optimisation

(4) Resonator profile

(5) Optimising DEER pulses

(6) Relaxation experiments

(7) Optimising DEER delays

(8) Initial DEER experiment (1–3 hours)

(9) Re-optimising DEER delays

(10) Final long DEER measurement for the remaining time

Whilst it is possible to create an automated protocol that requires no inputs, some basic information on the sample and the intended measurement improves parameter choice:

• Maximum measurement time: this is the maximum amount of time available to measure this sample. Providing this ensures that the best measurement is done within the available time. The protocol stops early if this measurement time is not required.

• Temperature: under normal conditions this is not required, however SRT optimisation (see Section 2.3) differs between room temperature and low temperature.

• Estimated labelling efficiency: it is useful to have an estimate of what fraction of spin labels are attached to a molecule that also has another spin label. This is used in Section 2.7.2. Labelling efficiency can be determined by continuous-wave EPR or mass spectrometry.^3^

• MNR priority: the operator can have very different priorities when measuring DEER, resulting in different target MNRs. If only the region of interest (ROI) of the distance distribution is necessary then a lower MNR ≈ 20 can be targeted, whilst to determine the distribution shape, a MNR > 100 is necessary. Additionally, an operator may intentionally measure a singly labelled molecule. In such a case, a target SNR is required, as the modulation depth is likely to be very small or zero. By default, an MNR target of 150 is recommended.

• Resonator centre frequency: at the moderate quality factor (50 ≲ *Q* ≲ 200) of an overcoupled resonator and in the presence of microwave (mw) reflection and standing waves in the bridge, fully automated analysis of tuning curves is challenging. Knowledge of the approximate centre frequency of the resonator makes this analysis much more robust. For most resonators, this value will change only little between samples with similar dielectric values.

### 2.1 Sample insertion

The initial step is sample insertion, which must comply with the specific requirements of the resonator and cryostat in use. For DEER experiments, which involve two-frequency EPR, it is crucial to employ an overcoupled resonator with a sufficient bandwidth to allow adequate separation of the two excitation frequency bands and maximisation of the overall measurement sensitivity.

If sample volume is not limited, oversized-sample *Q*-band resonators provide best sensitivity.^12^ Due to low conversion factors, such resonators require high incident mw power and thus expensive amplifiers. Lower-volume resonators with better conversion factors can be used with lower mw power at the cost of a moderate loss in concentration sensitivity.

The following steps of the protocol can be performed when the sample is inserted and its temperature has stabilised to the measurement temperature. The optimal temperature for nitroxide DEER experiments is around 50 K.^2,3^ Above this temperature, coherence lifetime shortens due to spatial dynamics, whereas below this temperature, slowdown of longitudinal relaxation requires a lower repetition rate of the experiment, which results in a net loss of signal-to-noise ratio (SNR) per time despite increased spin polarisation.

### 2.2 Field sweep

With the sample correctly inserted and the centre frequency of the resonator roughly identified, a preliminary EPR spectrum is measured by an echo-detected field sweep (EDFS). In this experiment, the Hahn echo amplitude is recorded as a function of the magnetic field. The centre field is estimated from the mw frequency by using the gyromagnetic ratio of a nitroxide radical. A smoothing spline is applied to the EDFS spectrum for noise reduction. The field corresponding to the maximum of the spectrum is used to calculate an improved gyromagnetic ratio, which includes any potential field offsets. This gyromagnetic ratio is used to determine the relation between magnetic field and mw frequency in further setup steps.

### 2.3 Shot repetition time optimisation

Next, the shot repetition time (SRT) is optimised at the spectral maximum. This optimisation is critically important, as it directly affects the SNR achievable in a given time. Too long SRT wastes measurement time. Too short SRT leads to saturation and reduces SNR. Even moderate saturation can bias the distance distribution if different conformers have different longitudinal relaxation times.^25^

**Fig. 3:**
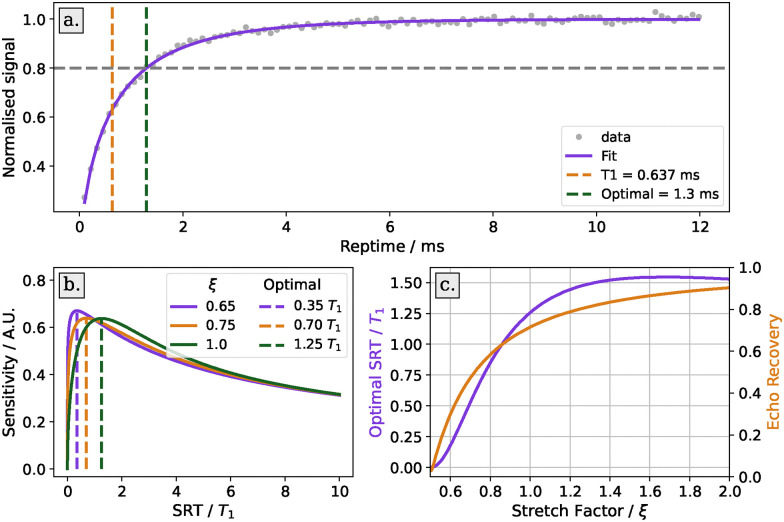
Optimisation of shot repetition time (SRT). Panel (a): SRT scan (grey dots) and its fit (red line) for the sample MBP 20/238 at 50 K on a Bruker ElexSys E580 spectrometer. The fit parameters are *T*_1_ = 1.55 ms and *ξ* = 0.70. The optimal recovery time is chosen such as to give a 80% recovery. Panel (b): illustration of the change of the optimal recovery time relative to *T*_1_ with changing stretching parameter *ξ*. Panel (c): the effect of the stretching parameter upon the optimal SRT and its corresponding echo recovery.

The optimal SRT is determined through an SRT scan. This is a series of Hahn echo experiments with increasing SRT. The result of a normalised SRT scan can be fitted with a single stretched-exponential recovery curve, *A*(1 − e^−(SRT/*T*_1_)^*ξ*^^). The parameters are the longitudinal relaxation time *T*_1_, a stretching factor *ξ* and an amplitude *A*. An example of this is shown in Fig. 3a.

From the estimated *T*_1_, the optimal SRT is calculated by maximising the function1
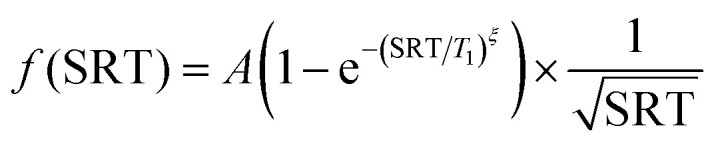


For a stretching factor (*ξ*) of 1 the maximum occurs at 1.3*T*_1_ which corresponds to a 75% signal recovery, and for *ξ* = 0.75 the maximum occurs at 0.7*T*_1_, which corresponds to ≈53% signal recovery What can be seen from the plot of sensitivity (Fig. 3b) against SRT is that underestimating the SRT leads to a rapid drop in sensitivity whilst overestimating SRT only leads to a small reduction in sensitivity. Additionally, some samples are heterogeneous, leading to a distribution in *T*_1_.^25^ With this limitation in mind, we do not recommend optimising the SRT for greatest sensitivity unless it is known that the sample is homogeneous. Instead, we follow the recommendation, set out in the community white paper, to set the SRT such that there is 80% signal recovery.^3,26^ This typically corresponds to a value around 2.5–3.5 ms for nitroxide samples at 50 K. With a *ξ* = 0.75 this corresponds to SRT = 1.9*T*_1_ and a 9% reduction in sensitivity against the ideal.

If a nitroxide label is measured at room temperature, then the optimal SRT is so short (around 200 µs) that an SRT scan is experimentally not possible. In this case, the SRT often needs to be extended to satisfy the amplifier maximum duty cycle limit.

### 2.4 Resonator profile

Next, the resonator profile is determined. This specifies the mw field amplitude *B*_1_ or, equivalently, the nutation frequency *ν*_1_ ∝ *B*_1_ as a function of mw frequency. The resonator effectively behaves as a filter for both excitation and detection and thus its profile determines how the excitation bands of observer and pump pulses should be set and at which frequency detection is most sensitive. Additionally, when using frequency-chirped pulses it is important to correct the waveform to obtain the intended uniform excitation profile.^28,29^ If the frequency dependence of all other elements of the excitation chain is much weaker than the one of the resonator mode, the resonator profile is equivalent to the transfer function *H* of the excitation chain and the forward reflection coefficient (*S*11).

The resonator profile is measured directly at the electron spins through a frequency-swept nutation experiment.^30^ In this experiment, the electron spins are nutated by a monochromatic high-power pulse of varying length. After a time delay greater than the phase memory time *T*_m_, the longitudinal electron magnetisation is detected by a Hahn echo using pulses of equal amplitude. This experiment is performed throughout a range of mw frequencies spanning the resonator mode. For best sensitivity, the magnetic field is adjusted with the mw frequency in order to always satisfy the resonance condition at the maximum of the nitroxide EPR spectrum. For *Q*-band operation, a 200–400 MHz frequency range is typically required, with measurement points spaced less than 40 MHz apart to ensure adequate resolution.

**Fig. 4:**
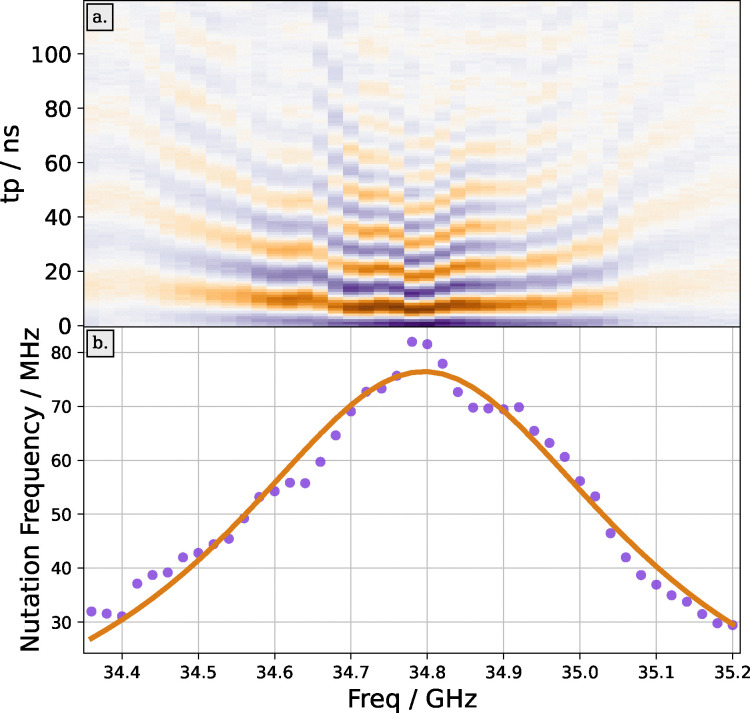
Determination of the resonator profile by nutation frequency measurements. Panel (a) shows the signal of an echo-detected nutation experiment as a function of mw frequency and pulse length. Panel (b) displays the dependence of nutation frequency *ν*_1_ on mw frequency. Experiments were performed with the MBP 20/238 protein test sample in a pent-loop-gap resonator.^27^

To reduce the resulting two-dimensional frequency-swept nutation data (Fig. 4) to a one-dimensional resonator profile, we must extract the nutation frequency at each mw frequency. Theoretically, the transient nutation signal for a resonance line much broader than the excitation bandwidth is given by:2
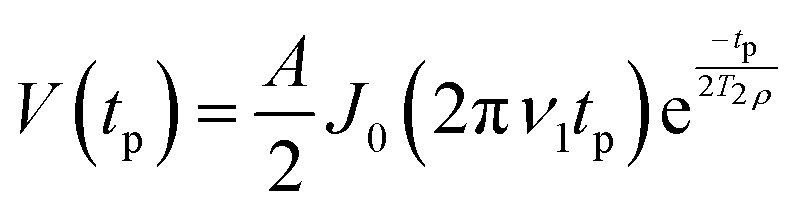
where *A* is the amplitude, *t*_p_ the length of the nutation pulse, *J*_0_ the zeroth-order Bessel function, *ν*_1_ the nutation frequency, and *T*_2*ρ*_ the decoherence time of the electron spins during mw irradiation.^4,31^

Previously, the nutation frequency was extracted by finding the maximum value of the magnitude of the Fourier transform of the nutation profile.^30^ We have found that in measurements with a low SNR this approach struggles to extract the correct frequencies.

Instead, we have tested determination of the nutation frequency by fitting the time-domain signal with either a decaying Bessel function (eqn (2)) or with a decaying cosine function,3
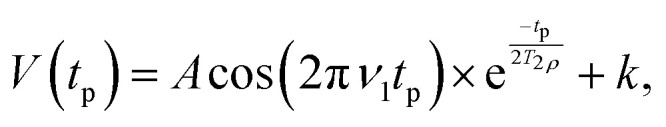
where all terms have the same meaning as in eqn (2). A comparison of fitting between a Bessel and a cosine can be seen in Fig. S1 in the SI. The decaying cosine both provides a better fit and leads to more robust fitting. It is likely that the cosine function fits better because we are measuring on the peak of the nitroxide spectrum where the assumption of a linewidth much larger than the excitation bandwidth is not fully valid.^4,31^

Nonetheless, even with a decaying cosine function, some nutation traces are difficult to fit. These poor-quality fits are discarded. To this end, we evaluate the coefficient of determination *R*^2^ of the fit, and discard all traces where *R*^2^ < 0.5.

Once the 1D resonator profile (shown in Fig. 4b) has been extracted, it can be fitted. In most cases the resonator profile will be modulated with reflections and standing waves from the mw bridge. Whilst these reflections can be important, for our purposes it suffices to fit the profile with a Lorentzian function,4
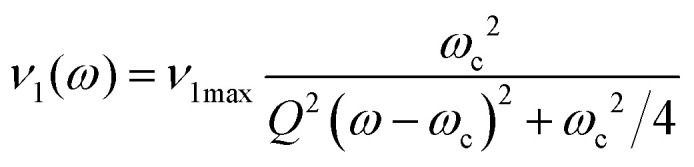
where *ω*_c_ is the resonator frequency, *ν*_1max_ is the maximum nutation frequency, and *Q* is the (loaded) resonator quality factor.

If the determined resonator central frequency differs from the initial one by more than 100 MHz, then the resonator profile measurement is repeated with the new centre frequency so that both sides of the resonator profile are measured, and a new field sweep is measured with the updated resonator frequency.

### 2.5 Optimising DEER pulses

In previous work, the frequency of DEER pulses was mostly selected by rules of thumb which were appropriate for rectangular pulses. In the recent community white paper it was stated that the separation should be in the range 80–100 MHz for rectangular pulses with lengths in the 8–32 ns range.^3^ This rule of thumb does not consider usage of frequency-swept pulses nor the specific resonator profile. The optimal conditions can vary depending on the *Q*-value of the resonator and the available mw field amplitude *ν*_1_.

Here we set out a generalised approach for determining the frequencies of observer and pump pulses based on both the measured EPR spectrum and resonator profile. This method is demonstrated for a range of common pulse shape combinations, both monochromatic and frequency-swept. Pulse excitation profiles are simulated using a two-level approach previously demonstrated in ref. 32 and 33 and more recently implemented in EasySpin.^34^ These excitation profiles take into account the transfer function *H* calculated from the resonator profile.

For simplicity, we optimise only the dominant dipolar pathway. The single-pathway DEER signal *V*(*t*) is given by

5 *V*(*t*) = *V*_0_[(1 − *λ*) + *λK*(*t*,*r*)*P*(*r*)]*B*(*t*),

where *λ* is the fractional modulation depth, *K* is the DEER kernel, *P* is the distance distribution and *B* is the background function from intermolecular dipolar coupling.^21^

In this single-pathway case, DEER sensitivity can be expressed as the product of two factors: the proportion of spins that are exclusively observed and the proportion of spins that are exclusively pumped that contribute to the measurement.^35^ The absolute sensitivity is also proportional to the total number of spins, but we do not consider it here. Instead, we maximise the modulation-to-noise ratio (MNR), which is given by the product of the fractional modulation depth (*λ*) with the signal-to-noise ratio (SNR), SNR = *V*_0_/*σ*, where *σ* is the standard deviation of Gaussian white noise:6
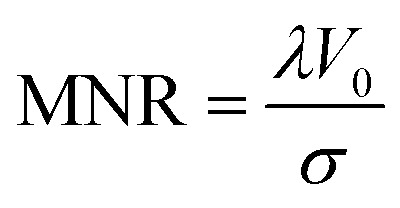


Therefore, we need to maximise the product of the modulation depth and the signal *λV*_0_ as the noise level *σ* does not depend on our control parameters. The noise level optimisation is dependent on the detection filter bandwidth used and is therefore instrumentation dependent. Where a matched filter is not possible, the echo integration width should be set such that it is twice the excitation (
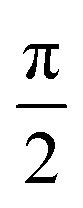
 pulse length).^36^

**Fig. 5:**
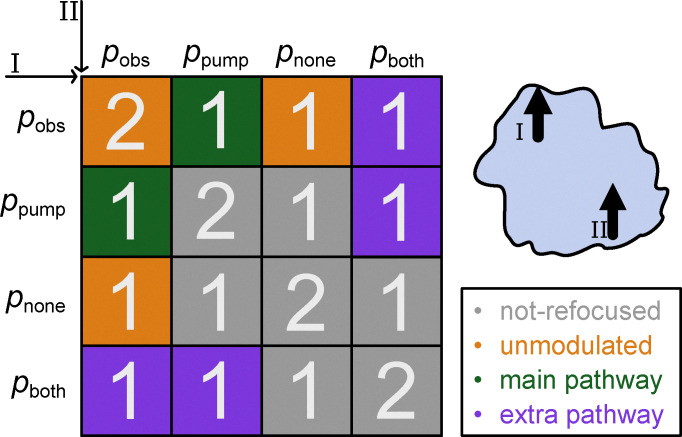
The matrix shows how the signal is affected by the probabilities that either spin I or spin II are exclusively observed (obs) or pumped (pump). Only if one of the two spins (spin A) is observed and the other spin is pumped (spin B) is the main-pathway modulated signal detected (green). If a spin is both pumped and observed then it forms an extra pathway (purple). If neither spin is pumped and one or more spin is observed than an unmodulated (background) signal is detected (orange) otherwise the signal is not refocused at detection time (grey). The white numbers correspond to the scale of the signal, 2 if both spins are the same and 1 otherwise.

The fractional modulation depth *λ* in the range between 0 and 1 is given by the fraction of the spin packets experiencing dipolar modulation (green in in Fig. 5) with respect to all spin packets that are refocused (purple and orange). Non-refocused spins do not contribute to the signal. In Fig. 5, it can be seen that the probability of a spin being modulated or unmodulated is given by, respectively,

7 *p*_mod_ = 2*p*_pump_*p*_obs_

8 *p*_un-mod_ = 2*p*_obs_*p*_none_ + 2*p*_obs_^2^.

Thus, the fractional modulation depth *λ* is given by9
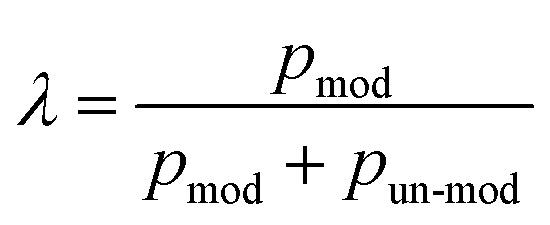
and for the total signal *V*_0_ we have

10 *V*_0_ ∝ *p*_mod_ + *p*_un-mod_.

For the MNR we thus have11
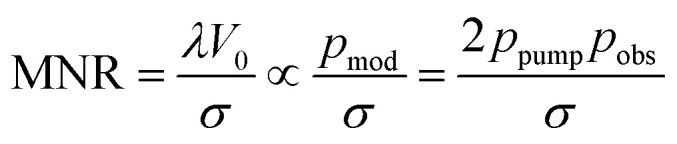
Where *p*_pump_ and *p*_obs_ represent the probabilities that a spin is exclusively pumped or observed respectively.12
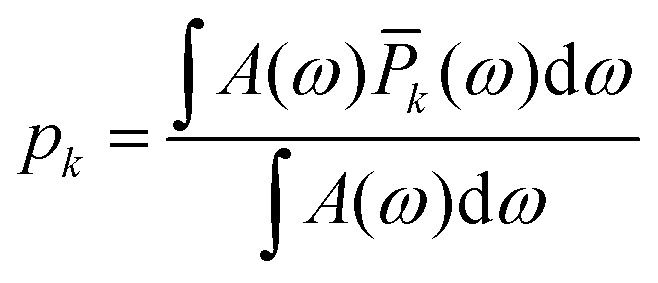
where *A*(*ω*) is the EPR spectrum and *P̄*_*k*_(*ω*) is the probability profile that a spin is exclusively pumped or observed. *P̄* is calculated from the product of the pulse excitation (either coherence generation or inversion) profiles minus any pump-observer overlap and detailed in SI S2.1. The multi-pathway effects from any overlap are assumed to be small and are not considered.

We optimise the MNR for optimal pulse position by maximising the functional

13 *F*(*ω*_p_, *ω*_o_, *ω*_A_) = 2*p*_pump_(*ω*_p_)*p*_obs_(*ω*_o_)

as a function of the pump frequency *ω*_p_, the observer frequency *ω*_o_ and the nitroxide spectrum maximum frequency *ω*_A_.

Ideally we would include how the excitation profiles are affected by the pulse bandwidth, pulse length or other additional pulse parameters directly in our functional. The inclusion of these additional parameters is, however, not computationally feasible in a time of less than one minute that is reasonable for a setup procedure. Instead, we re-run the optimisation using several predefined pulse setups.

The specific resonator profile has a great effect on this optimisation procedure, however at the moment it is not feasible to perform fine optimisation. This would require that a high-quality resonator profile (tune picture) would be measurable in a matter of seconds and that the resonator had a motorised coupling screw.

The excitation pulse in the observer sequence is set to be always a rectangular pulse. We have chosen to only use rectangular observer pulses due to their simplicity and lower power than a Gaussian pulse.^37^ The cumulative observer pulse train profile suppresses any sidebands, nonetheless Gaussian pulses can also be used. The length of this pulse is set to 
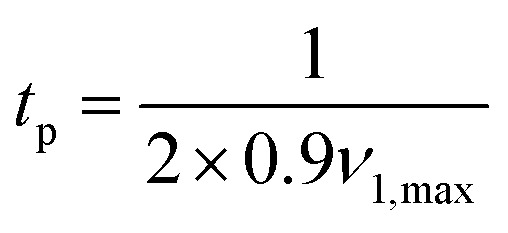
, keeping its bandwidth at 90% of the maximum nutation frequency *ν*_1max_. Should this length result in a pulse excitation bandwidth larger than 40% of the available bandwidth Δ*ν* (resonator or spectrum, whichever is narrower), then the pulse length is set to 
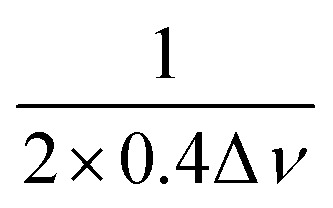
.

The refocusing pulses in the observer sequence are set to be the same shape as the excitation pulse but will require twice the amplitude as they have a flip angle of π.

On an AWG-equipped spectrometer a range of pump pulses are tested to see what is most suitable with the resonator. For other spectrometers only rectangular pulses are optimised. By default, we test monochromatic and linear-chirped rectangular pump pulses, however Gaussian and hyperbolic-secant are also possible. Our investigations showed that a hyperbolic-secant pulse was only beneficial when a low-*Q*, high-*B*_1_ resonator was used which is highly uncommon, see SI Fig. S2. If the pulse is rectangular or Gaussian, its bandwidth (*i.e.* pulse length or full-width-half-maximum respectively) is set to equal the one of the excitation pulse. For the frequency-swept pulses, the sweep bandwidth (BW) is set to

14 BW_pump_ = min([BW_resonator_, BW_spectrum_]) − BW_excitation_.

When using frequency-swept pulses, we use the lower bound of the distance region of interest (ROI) to verify that the length of the pulse does not cause artificial broadening of the distance distribution.^38–40^ The pump pulse length is then set by15
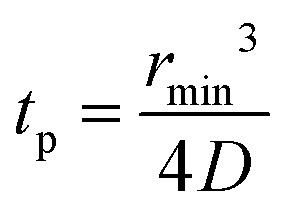
where *t*_p_ is the pump pulse length, *r*_min_ is lower bound of the ROI, and *D* is the dipolar constant, 52.04 MHz nm^3^ for a nitroxide spin label. During the initial DEER experiment we do not know *r*_min_ and cannot apply this criterion, however as we are only aiming to identify if distances are present at this point in setup, additional broadening in the distance distribution is not an issue. Additionally, the pump pulse length is limited to 256 ns or 10% of the dipolar evolution time, *τ*_evo_, whichever is less. In the case of a very broad distribution, where only the rough shape can be determined, additional broadening is not a concern. If additional broadening is a concern to the operator, then the measurement should be re-run with a restriction to rectangular pulses and the distributions compared.

The pump pulse giving the largest value of the functional eqn (13) is selected.

#### 2.5.1 Examples of optimal excitation conditions

To illustrate the result of such optimisation, we have simulated excitation profiles at the optimal pulse frequencies for a selection of common resonator and amplifier setups, which have been chosen because they closely resemble either the experimental conditions of application examples below or are commonly available instrumentation. The simulated resonators do not consider the effect of sample volume on the concentration sensitivity.

• Resonator 1: is modelled on our home-built pent-loop-gap resonators with a 1.6 mm sample tube paired with a 200 W TWT amplifier, this has the parameters *Q* = 70, *ν*_1,max_ = 70 MHz.^27^

• Resonator 2: is modelled on our oversized-sample *Q*-band resonators with a 3 mm sample tube paired with a 200 W TWT amplifier^41^ giving the parameters *Q* = 100, *ν*_1,max_ = 45 MHz. These parameters are similar to what can be expected with the smaller sample volume (1.6 mm) Bruker D2 resonator and a high-power TWT amplifier that is widely used.

• Resonator 3: resembles conditions for a moderate-power *Q*-band spectrometer, such as using a 50 W solid-state-power-amplifier with a Bruker D2 resonator. Here we have chosen parameters of *Q* = 120, *ν*_1,max_ = 25 MHz.

Excitation profiles for all resonators have been simulated with the same resonator central frequency, *ω*_c_ = 34.0 GHz. The modulation depth *λ* and optimisation functional *F* were calculated using eqn (9) and (13) respectively.

**Fig. 6:**
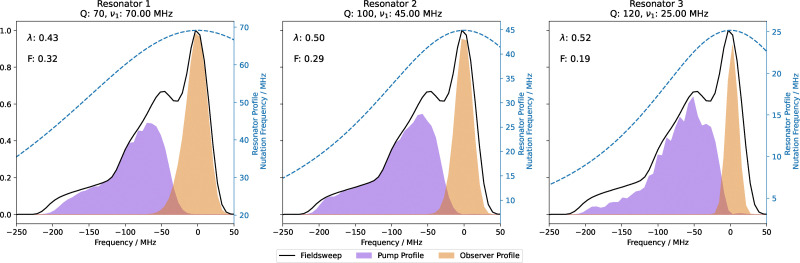
The optimal pump and observer excitation frequency profiles are shown with respect to the EPR spectrum for different resonators. Additionally, the modulation depth (*λ*) and sensitivity (*F*) are given. The pulses were optimised for four-pulse DEER. Resonator 1 was optimised to have 8 ns rectangular excitation and refocusing pulses paired with a 206 ns, 180 MHz linear-chirped rectangular pump pulse centred at −115 MHz. Resonator 2 was optimised to have 12.5 ns rectangular excitation and refocusing pulses paired with a 206 ns, 200 MHz linear-chirped rectangular pump pulse centred at −119 MHz. Resonator 3 was optimised to have 23 ns rectangular excitation and refocusing pulses paired with a 206 ns, 220 MHz linear-chirped rectangular pump pulse centred at −116 MHz. All excitation pulses were centred at 0 MHz.

The optimal pulse excitation profiles for these resonators are shown in Fig. 6. All three samples used linear-chirped rectangular pulse, however in the case of resonator 3 the bandwidth was 220 MHz in contrast to 200 MHz, where a narrower observer profile led to a higher estimated modulation depth but significantly lower sensitivity.

Resonator 1 has the highest sensitivity, followed by resonator 2 (10% lower) and resonator 3 (40% lower). Resonator 1 was more sensitive than resonator 2 even though the modulation depth is estimated to be lower, due to the higher bandwidth (shorter) observer pulses. The substantial sensitivity reduction in resonator 3 compared with 1 was primarily due to the lower bandwidth (longer) rectangular observer pulse, leading to less observed spins.

### 2.6 Relaxation experiments

Understanding the spin relaxation of the specific sample is not only important for determining the optimal DEER inter-pulse delays, but also for understanding the local environment of the sample and how it could be optimised to further increase the maximal inter-pulse delay. Longer inter-pulse delays enable a longer dipolar evolution time, and therefore detection of longer distances, better distribution shapes, and better background identification.

**Fig. 7:**
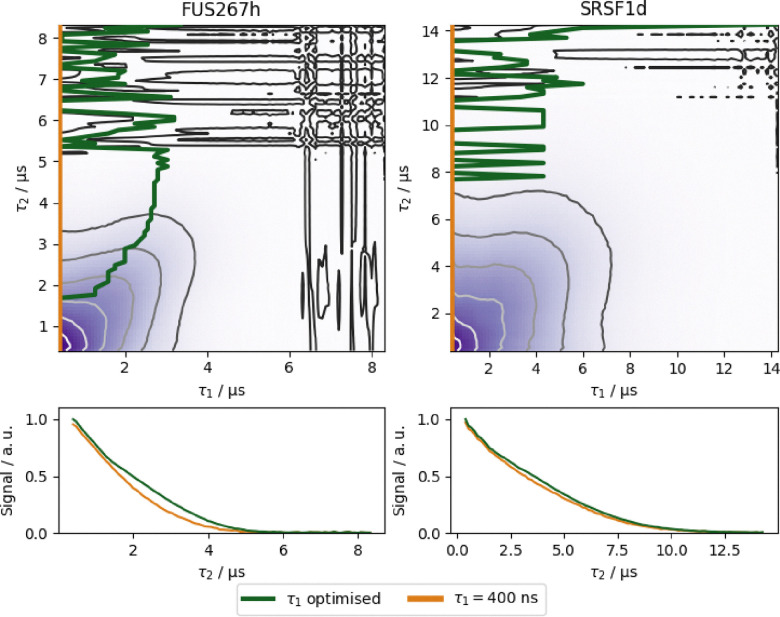
The 2D refocused echo experiment is shown for both the FUS267 and SRSF1 proteins with a protonated and deuterated buffer, respectively. The red colour shows the common *τ*_1_ = 400 ns position, and the green colour the optimised *τ*_1_. In the bottom sub-figures, the signals for these *τ*_1_s are compared. In the protonated FUS sample there is a clear signal advantage of using an optimised *τ*_1_, extending the maximum measurable *τ*_evo_, however in the SRSF1 sample there is no significant difference.

In this protocol we aim to select the optimal pulse sequence so we need to investigate the relaxation behaviour of both the four-pulse and five-pulse DEER sequences. Traditionally, most published DEER data has been accompanied by Hahn-echo relaxation data. However, relaxation affects the Hahn echo differently than the refocused echo that is used in either four-pulse or five-pulse DEER.^42^ For four-pulse DEER, it has been recently demonstrated that the optimal *τ*_1_ can be longer than 400 ns.^42^ Therefore, the refocused echo is recorded as a function of both inter-pulse delays *τ*_1_ and *τ*_2_. Examples for the SRSF1 and FUS267 samples are shown in Fig. 7 and the whole data set in SI Fig. S3. From these 2D data, we identify the maximum signal for each *τ*_2_, shown by the green line. This 1D trace gives the optimal *τ*_1_ for a given *τ*_2_.

Measuring a 2D experiment is, however, quite time-consuming and in cases where the matrix is deuterated a short *τ*_1_ is often optimal. This is especially true as five-pulse DEER is optimal for most samples. In our proposed protocol, we only measure a 2D refocused echo experiment when we are optimising a four-pulse DEER sequence from the start. Otherwise, a 1D refocused echo relaxation experiment, where *τ*_1_ = 400 ns is sufficient for most samples and accommodate long pump-pulses.

For five-pulse DEER where the delays satisfy the Carr–Purcell-2 condition *τ*_1_ = *τ*_2_, we measure the Carr–Purcell decay.

**Fig. 8:**
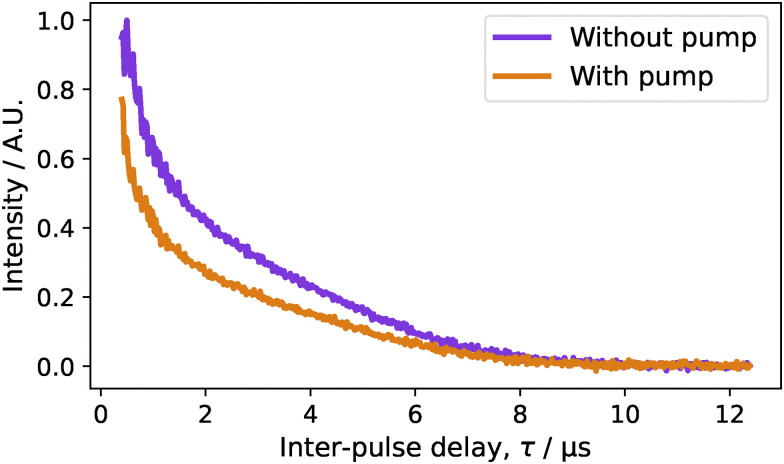
A comparison on the Carr–Purcell-2 decay for the 20 µM MBP 238/275 sample with and without the presence of a 12 ns pump pulse set to −80 MHz from the 12 ns observer frequency which is placed on the spectral maximum. The pump pulse is applied at the maximum of the primary echo/maximum of the DEER trace.

If the spectrometer permits, the relaxation measurements should use the same pulse setup as we aim to use for DEER so that the sensitivity is most comparable. Since the pump pulse can also enhance relaxation due to instantaneous diffusion, it should be included if possible. This pump-pulse-induced echo amplitude reduction effect has not been widely reported on nitroxide spin labels, but is substantial for gadolinium-based spin labels.^38,43–45^ Across our whole sample set a global echo reduction effect was observed. An example for the MBP 238/275 sample is shown in Fig. 8, and all the data are shown in the SI Fig. S4.

### 2.7 Optimising DEER delays

A crucial aspect of the optimisation of a DEER experiment is the positioning of the pulses in the time domain. This includes the static inter-pulse delays (often referred to as *τ*_1_, *τ*_2_ and *τ*_3_ or as *d*_1_, *d*_2_ and *d*_3_) as well as the moving pump pulse delay (*t*). For the moving pump pulse, we need to determine the optimal start value *t*_min_ and the time step Δ*t*. In this paper all time delays refer to centre-to-centre time, and not start-to-start. This has been done to better handle both short rectangular pulses and long frequency-swept pulses.

#### 2.7.1 Selection of the time step

The minimal detectable distance is limited by the pump pulse time step due to the Nyquist criterion down to a lower limit imposed by the excitation bandwidth of around 1.5 nm.^11^ A safety margin is added to the Nyquist criterion of 85% and as the DEER Pake pattern has shoulders at twice the dipolar frequency we use a factor of 4 instead of 216
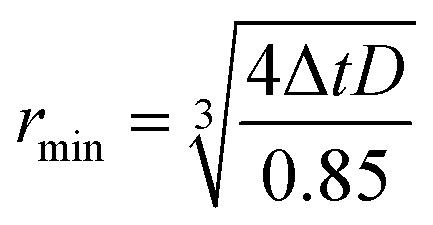
where *r*_min_ is the minimum detectable distance, Δ*t* is the time step and *D* = 52.04 MHz nm^3^ the nitroxide radicals dipolar constant.^2,46^

Since all DEER data is fitted with a least-squares based algorithm, oversampling the data by reducing Δ*t* to be shorter than is required by eqn (16) should have the same effect on fit quality as increased averaging. We have analysed the effect on fit quality of both over- and undersampling different simulated distance distributions and compared how well the fit is able to reproduce simulated data, which is shown in SI Fig. S5C.

A consequence of oversampling is that the noise amplitude is higher than would be achieved by performing a higher number of averages with a longer time step. However, by defining a minimum distance in data analysis, we apply in effect a low-pass filter to the data that suppresses high-frequency noise. The consequence is that MNR and SNR criteria are meaningful only if the time step and minimum distance are also specified. On the other hand, undersampling can result in a suppression of short distances according to eqn (16). The quality of a DEER experiment cannot be judged by its SNR or MNR alone, and rules preventing undersampling are essential.

As a consequence, the time step can be as short as the spectrometer hardware allows, which is between 0.1 and 2 ns. This way aliasing of high-frequency noise into the frequency region of interest is safely excluded. However, such a short time step significantly increases the number of points per scan and subsequently the measurement time per scan. For strong-signal samples this may increase the measurement time. Normally, it is advantageous to measure more than one scan so that slow processing noise sources (*e.g.* temperature fluctuations) are averaged out.

However, on many modern arbitrary waveform generator (AWG) based spectrometers there also exists an AWG memory limit, which provides an upper limit on the number of points in a scan. This depends on the particular instrument. Therefore, we lay out a set of rules that can be used to set the time step, where if possible the time step should be sufficiently short for covering dipolar frequency at the minimum distance of 1.5 nm accessible by DEER.^11^ Where possible, we suggest that Δ*t* = 8 ns which corresponds to a lower distance limit of 1.2 nm. If this way the experiment does not fit into the AWG memory then Δ*t* can be increased. If Δ*t* > 16 ns (1.58 nm) then there is a risk of distortions in the short-distance regime; if such short distances are not expected then this longer time step can be used. However, in the unusual situation where the operator expects both very short (<1.6 nm) and very long distances, then two separate DEER measurement are required, one with a short Δ*t* and the two data sets should be globally fitted. This unusual situation is beyond the scope of this automated protocol. This situation may benefit from non-uniform sampling (NUS) or larger-memory AWGs or continuous data streaming between the control computer and the AWG during the experiment. There is initial proof-of-concept work on using NUS with DEER, however this approach has not yet reached maturity.^47–49^ As most commercial and home-built spectrometers do not support NUS, we leave such development as an extension for the future.

#### 2.7.2 Selection of inter-pulse delays

One of the key parameters used for the selection of the DEER pulse sequence is *τ*_evo_, so we calculate the optimal inter-pulse delays for both four- and five-pulse DEER. *τ*_evo_ determines the upper distance limit that can be interpreted. An established good rule of thumb is^2,50^17
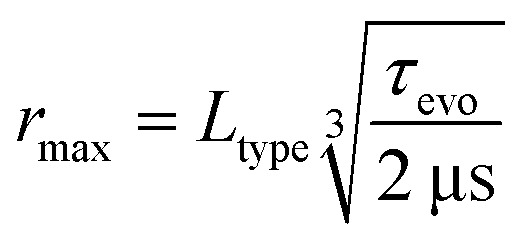
where the value of *L*_type_ depends on the desired information content: *L*_type_ = 6 nm for identifying the presence of distances, *L*_type_ = 5 nm for determining the mean distance, *L*_type_ = 4 nm for measuring the distribution width, and *L*_type_ = 3 nm for resolving the distribution shape.

For many samples, the available measurement time falls short of what is needed for optimal results, necessitating a careful balance between the required MNR and *τ*_evo_. If the maximum distance present is known (for instance, following an initial DEER measurement) and the appropriate *τ*_evo_ can be achieved within the available measurement time, this value is implemented. More commonly, however, we strive to maximize *τ*_evo_ and consequently *r*_max_.

Using the relaxation data measured in Section 2.6, we can calculate the optimal inter-pulse delays for both the four-pulse and five-pulse DEER sequences. For four-pulse DEER *τ*_evo_ ≈ *τ*_2_ and for five-pulse DEER *τ*_evo_ ≈ 2*τ* and the normalised (to one) signal decay is denoted as *Ṽ*(*τ*_evo_).

*Ṽ* is then further normalised by the total number of acquisitions (*N*_acq_, which combines the number of averages, shots, and phase cycle steps) and the noise level (*σ*), to give the SNR as a function of *τ*_evo_. The total SNR achievable with a given measurement time (*T*) is18
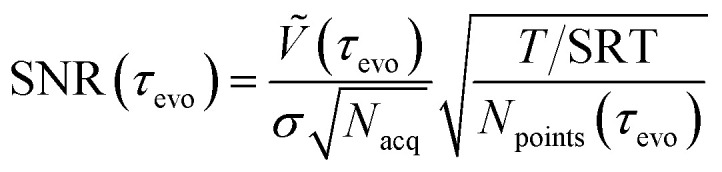
where SRT is the shot repetition time and *N*_points_(*τ*_evo_) is the number of data points per scan, for a uniformly sampled trace *N*_points_(*τ*_evo_) = *τ*_evo_/Δ*t*.

The optimal *τ*_evo_ value can then be found as the root of eqn (18) for a specific SNR and *T*. In circumstances where there is more than one root, the largest root is taken.

The necessary SNR is given by SNR = MNR/*λ*. Before the initial DEER measurement, it is only possible to estimate the modulation depth. Eqn (9) estimates a theoretical modulation depth for this setup; the real modulation depth will likely be lower than this as this equation assumes a 100% labelling efficiency. Often the operator will have an understanding of the labelling efficiency from continuous-wave EPR experiments and should then provide this value. After the initial DEER measurement the actual modulation depth is known.

The identification of the optimal *τ*_evo_ found from eqn (18) can be susceptible to noise, and since a higher-than-predicted SNR is a better outcome than a lower-than-predicted SNR, we calculate and use a lower bound for *τ*_evo_ in further setup. The upper and lower bounds are calculated by *Ṽ*_ub_ = *Ṽ* + *σ* and *Ṽ*_lb_ = *Ṽ* − *σ*, where *σ* is the standard deviation.

**Fig. 9:**
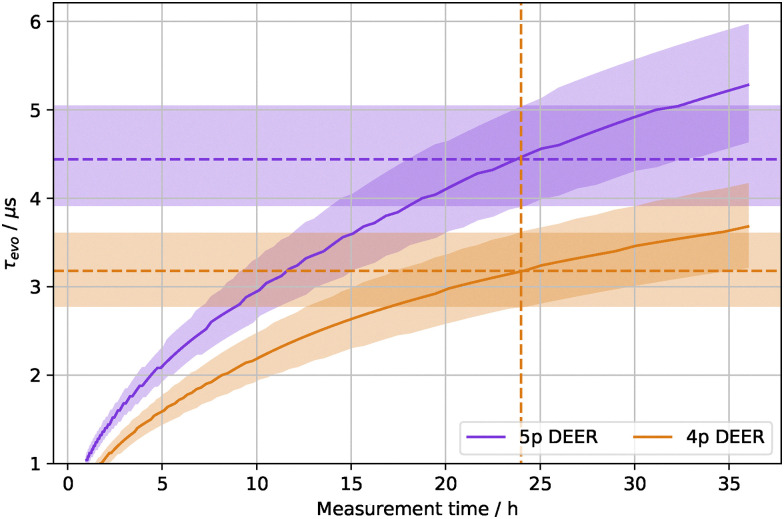
The predicted maximum achievable dipolar evolution time *τ*_evo_ as a function of measurement time for a modulation-to-noise ratios (MNRs) 150, assuming a modulation depth of 0.6 for the MBP 20/238 sample and using the relaxation data in Fig. 12e. This is shown in red for five-pulse DEER and in turquoise for the four-pulse DEER. Dashed lines and corresponding uncertainty bands correspond to a measurement time of 24 h.

It is important for the operator to receive feedback from this optimisation and see why the choice has been made so they can determine if changing the available measurement time makes sense. To do this, we solve eqn (18) for a range of measurement times and a given SNR. This allows us to create a figure showing *τ*_evo_ as a function of *T* for a given SNR. An example is given in Fig. 9. Experienced operators can thus use additional knowledge and experience with a sample to make a better choice than can be made by a fully automated procedure.

For the five-pulse DEER sequence, we have an additional inter-pulse delay, *τ*_3_, between the first (static) pump pulse and the first refocusing pulse. This parameter effectively adjusts the refocusing time of the dominant five-pulse pathway and should be set to be as small as possible whilst still keeping pulses sufficiently separated and allowing the necessary *t*_min_. A value of *τ*_3_ = 300 ns is appropriate for most pulse setups, and maintaining necessary pulse separation for frequency swept pulses.

#### 2.7.3 Selection of the DEER experiment

In cases where contributions at long distances are expected, it is advantageous to measure with the longest feasible *τ*_evo_. Depending on the sample, the four-pulse or the five-pulse DEER experiment may be better suited for that. We have developed a simple algorithm for determining which sequence to use.

At this point, we have already calculated the optimal inter-pulse delays for each of the DEER experiments and subsequently their respective maximum *τ*_evo_. We use this information to determine the best experiment. Four-pulse DEER is selected over five-pulse DEER if it can give similar or better information since it has fewer dipolar pathways to consider in data analysis, making the analysis more robust. Four-pulse DEER is preferred in the following situations:

(1) If four-pulse DEER can achieve a longer *τ*_evo_ in the same measurement time;

(2) If four-pulse DEER can give a similar (within 10%) signal amplitude at the same *τ*_evo_; and

(3) If the mean dipolar frequency, *ω*_dd_, is similar to *τ*_1_^−1^, since then it can be challenging to fit the five-pulse DEER trace due to the overlap between the two main pathways. This criterion is only applicable if the ROI has been determined or is known.

If we already have a target *τ*_evo_ which is achievable in the available measurement time (*e.g.* for the final DEER measurement) then only considerations 2 and 3 are relevant.

#### 2.7.4 Selection of *t*_min_

**Fig. 10:**
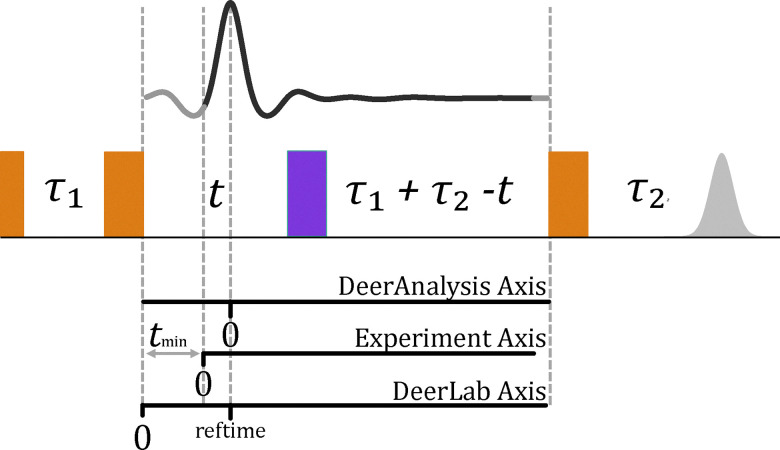
A comparison of the different dipolar axes definitions used in DeerLab, DeerAnalysis and during experiments.

Traditionally, optimisation of *t*_min_, historically referred to as zero-time or dead-time (See Fig. 10), has been overlooked. Of the seven labs that measured samples for the community white paper,^3^ only two explicitly specified *t*_min_, and it is not defined in the recommended pulse parameters. Many popular dipolar EPR analysis tools, such as DeerAnalysis and DEERNet^46,49,51^ use data points prior to the first maximum to identify the maximum, but then remove all these data points. With this approach, it would be detrimental to have a *t*_min_ greater than about 100–200 ns. Some dipolar fitting tools, such as DeerLab, can fit both sides of the symmetric dipolar function.^22^ We have investigated this through simulating DEER data with different *t*_min_ and comparing their fitted distance distributions to the simulated distributions, see SI Section S5. The conclusion is that *t*_min_ = 150 ns is sufficient for all situations.

### 2.8 Initial DEER experiment

Before measuring a high-quality DEER trace, it is crucial to determine the range of distances present in the sample, which we refer to as the region of interest (ROI). The operator may not have a good estimate of the ROI from prior knowledge. The ROI can be robustly identified with a much lower MNR than is necessary to determine distribution shape and hence in a relatively short measurement time, see SI Fig. S6a and S6b. We aim for an MNR of 20 in 2 hours of measurement time for such an initial measurement. However, the experiment is allowed to run for a maximum of 4 hours if needed. Based on this requirement, we select the optimal inter-pulse delays as outlined in Section 2.7.2. Normally, this measurement is performed by a five-pulse DEER sequence (Section 2.7.3) to maximise MNR.

We calculate the MNR during the measurement through a simple two-pathway fit (or a single-pathway in four-pulse DEER) with DeerLab, which is done after each scan and concurrently to the following scan. Once the requested MNR has been achieved, the measurement is stopped and the ROI is determined.

For samples with unexpectedly low modulation depth, the MNR of 20 may not be reached in a reasonable time of 4 hours. If the MNR is already greater than 10, then the protocol can continue. Otherwise, the measurement is stopped and the initial DEER experiment is repeated with a lower estimate for labelling efficiency.

Once the experiment has finished we then run a fully automated data analysis protocol using DeerLab that is set out in the SI Section S7.

#### 2.8.1 Calculation of distance region of interest

The accurate determination of the distance region of interest (ROI) requires a smooth distance distribution without substantial artefact peaks. To achieve this, we analyse the DEER trace using the compactness criterion and multiple pathways.^21,52^ The compactness criterion improves identifiability of the background. We found that the use of a parametric distance model, such as a Gaussian, is too unstable for the implementation in an automated algorithm. This instability primarily stems from the need to set appropriate starting parameters for the fit.

To calculate the ROI, we fit a Gaussian to the distance distribution originally determined by a non-parametric fit. The Gaussian mean value (*x̄*) and standard deviation (*σ*) are used to define the ROI.19
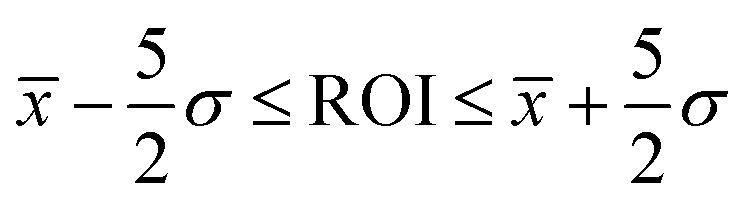


We tested this method against a cumulative integral-based approach and found that it is more robust against any residual artefacts.

### 2.9 Re-optimise DEER parameters and final DEER experiment

With the ROI determined, we can now determine the optimal parameters for the high-quality DEER measurement. The ROI primarily affects the inter-pulse delays. Ideally, we wish to determine the shape of the distribution and so the optimal *τ*_evo_ can be determined from eqn (17) using a *L*_type_ = 3 nm. However, electron spin decoherence is often too rapid for achieving a sufficiently high MNR at this *τ*_evo_. In such instances, we use the longest *τ*_evo_ that is expected to achieve an adequate MNR in the specified measurement time. This value is determined as described in Section 2.7.2. Here we do not need to estimate the modulation depth, as it is known from analysing data from the initial DEER experiment.

Now that we have identified the ROI and a new *τ*_evo_, we must check that the pump pulse length does not cause broadening using eqn (15) and the requirements set out in Section 2.5. If the pump pulse changes then it will require that the pulses be re-setup.

All other parameters are determined the same way as for the initial DEER experiment. The following high-quality experiment should be run for the remaining time in the allocated measurement period or until a sufficiently high MNR is achieved, typically around 150, see SI Fig. S6a. This is optimal for most applications where the shape of the distance distribution needs to be inferred. However, for challenging samples an operator may choose to prioritise a longer *τ*_evo_ and subsequently a higher maximum distance over MNR. If the measurement reaches this threshold in a specified absence period of the operator, for instance during the night, it should continue to improve data quality, unless automated sample change is implemented.

## 3 autoDEER

The above protocol was implemented into a Python-based software package, autoDEER. This software package enables push-button automated DEER measurements by operators with minimal EPR training. Through the use of a separate pulse EPR automation and pulse programming library, PyEPR, autoDEER can operate on a variety of commercial and home-built spectrometers. In particular, the current version of autoDEER supports the Bruker E580 spectrometer in both its AWG and MPFU configurations, which is currently the spectrometer most in use for such measurements.

**Fig. 11:**
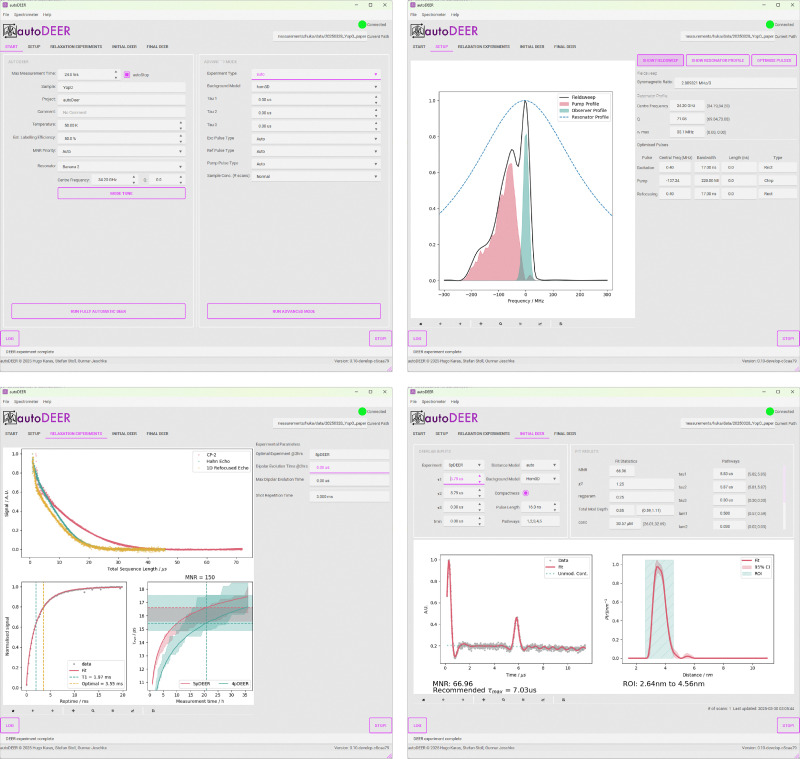
Screenshots of the autoDEER graphical user interface. The panels represent steps of the algorithm in a left-to-right top-down order. The top-left panel shows the operator input startup screen. If the operator presses the “fully automated” button, then the default automated settings are used. Otherwise the operator can specify more detailed options such as inter-pulse delays and sequence type. In the top-right panel the EDFS is shown along with the resonator profile fit and the excitation profiles of the optimised pulses. In the bottom-left panel the relaxation data is shown and in the final bottom-right panel the initial DEER measurement is shown.

The autoDEER package includes a simple-to-use graphical user interface (GUI) that provides rapid feedback on the progress on the measurement in the form of publication-quality plots created at each step. Automated DEER analysis as detailed in SI Section S7 is performed during the experiments, so the operator can see the latest fit as the experiment is progressing. Screenshots of the GUI are shown in Fig. 11.

The operator only needs to input a minimum of information (as detailed in Section 2) on the intended measurement, sample, and resonator. The sample and project name are required so that all data can be automatically saved in such a way that it can be autoclaimed by repository software such as LOGS.^53^

A configuration file is used for setting hardware-related parameters, such as the frequency range accessible by the spectrometer, and for customising the protocol, for instance, by providing the SRT recovery percentage.

In an advanced mode of autoDEER, expert operators can make their own parameters choices, for instance of the inter-pulse delays (*τ*_1_, *τ*_2_), the pulse shapes or select the DEER sequence, whilst still benefiting from the automated setup and analysis.

## 4 Materials and equipment

To demonstrate the reliability of the protocol, we tested it on a broad set of published samples ranging from simple short and rigid proteins to more complicated intrinsically disordered proteins, representing the diversity of samples that are encountered in practice. Both protonated and deuterated matrices have been tested along with a wide range of sample concentrations. Additionally, we tested the samples on both commercial and home-built spectrometers and a range of resonators.

### 4.1 Test samples

In this section, we detail the samples used in our paper to demonstrate the efficacy and versatility of our automated protocol.

#### 4.1.1 Sample 1: SRSF1

The serine/arginine-rich splicing factor 1 (SRSF1) has two RNA recognition motifs connected with a flexible linker. This specific sample has MTSL labels placed in each recognition motifs at sites Y37R1 and T169R1. Detailed sample preparation is described in a previous publication.^23^ The sample was filled into a 3 mm OD sample tube with a deuterated 1 : 1 (v/v) water : glycerol solvent to a final protein concentration of 15 µM. The sample was flash-frozen in liquid-nitrogen cooled isopentane and then stored in liquid nitrogen.

#### 4.1.2 Sample 2: FUS267

We also tested the RNA-binding protein fused in sarcoma (FUS), previously studied by Esteban-Hofer *et al.*^15^ The sample we investigated corresponds to the N-terminal domain (residues 1–167) of FUS protein in its dispersed state in 3 M urea. The labelled residues are A105R1 and G128R1. The samples had a protein concentration of 5 µM in agarose buffer (30 mM HEPES, 200 mM KCl, 0.5% (w/v) agarose, pH 7.3) at a temperature above the setting temperature of the gel and 35 µL was transferred to a preheated 3 mm OD sample tube, flash-frozen in liquid-nitrogen cooled isopentane and then stored in liquid nitrogen.

#### 4.1.3 Sample 3: PTBP1

The polypyrimidine-tract binding protein 1 (PTBP1) is an RNA-binding protein that was previously studied using DEER spectroscopy by Dorn *et al.*^5^ Here, we include the sample labelled at sites T109R1 and S475R1, in the presence of encephalomyocarditis virus internal ribosome entry site (IRES) RNA. The protein was fully deuterated and was diluted 1 : 1 (v/v) with d_8_-glycerol (Sigma Aldrich) and flash-frozen in liquid nitrogen to a final protein concentration of ≈50 µM. This sample was measured in a 3 mm OD capillary.

#### 4.1.4 Sample 4: YopO 599/624

The protein Yersinia outer protein O (YopO) has been widely studied in the recent community white paper.^3^ Here, we use the double mutant V599R1/N624R1 (sample B). The spin concentration is 50 µM, and the sample was prepared in a deuterated buffer before being diluted 1 : 1 with ethylene glycole-d_6_. It has been stored in its original 3 mm OD sample tube in liquid nitrogen. The dataset generated by lab A was used as a literature reference.

#### 4.1.5 Sample 5: MBP 20/238

Maltodextrin binding protein was previously studied by Tessmer *et al.*^24^ This sample is the spin-labelled construct L20R1/S238R1. A protein concentration of 13.5 µM in D_2_O with 20% d_8_-glycerol, 20 mM Tris at pH 7.5 and 120 mM NaCl was filled into a 1.6 mm OD sample tube.

#### 4.1.6 Sample 6: MBP 238/275

The second sample of maltodextrin binding protein is doubly labelled with bromo-MTSL (R7) at sites S238C and L275C. A protein concentration of 20 µM in the same buffer as sample 5 was filled into a 1.6 mm OD sample tube.

### 4.2 Equipment

The majority of all measurements in this paper were done using a home-built AWG-based EPR spectrometer at ETH Zürich.^28^ It uses a Bruker Q-band magnet with an Oxford Instruments cryostat. It is equipped with an Applied System Engineering 150 W TWT amplifier (>200 W power) and an Agilent (now Keysight) M8190A 12 GSa s^−1^ AWG operated at 8 GSa s^−1^. The signal is detected by an SP devices 12-bit ADQ32 digitiser operating at 4 GSa s^−1^ with a 2.5 GHz bandwidth. Only the in-phase component is detected and the quadrature component is calculated using the Hilbert transform. This is feasible because the transients are detected at a 1.5 GHz offset frequency and digitally down-converted to baseband.

This spectrometer is either equipped with both a home-built pent-loop-gap resonator with a 1.6 mm OD sample tube^27^ and a home-built oversized box resonator with a 3 mm OD sample tube.^12,41^ All samples in 3 mm OD sample tubes were measured with the home-built oversized resonator and all samples in 1.6 mm OD sample tubes were measured with the home-built pent-loop gap resonator.

To demonstrate the applicability of the automated protocol to a commercial instrument, the protocol was tested on a Bruker ElexSys-II E580 spectrometer at Bruker BioSpin in Ettlingen, Germany. This spectrometer was equipped with the SpinJet-II AWG, SpecJet-III digitiser, PatternJet-III and VAMP-III modules. A Bruker 50 W solid-state power amplifier was used in conjunction with the Q-band D2 dielectric resonator and 1.6 mm OD sample tubes. The spectrometer was controlled with a pre-release version of Xepr 2.9.

In contrast to the home-built spectrometer, implementation of the repetition time scan at the Bruker spectrometer features a reprogramming delay between points. This results in the recovery curves having longer rise times on the home-built spectrometer. The only effect on the protocol is that selected SRTs are longer than optimal at the home-built spectrometer resulting in somewhat lower sensitivity. This can be seen in SI Fig. S8, where SRT scans are compared between the home-built spectrometer and a Bruker E580.

## 5 Results

To test and evaluate both the protocol and the autoDEER software, we ran the automated protocol on our test sample set. The target measurement time was set to 24 hours for all samples. In all cases the estimated labelling efficiency was set to 100%, the default MNR priority of 150 was selected, and the resonator centre frequency was set appropriately.

**Fig. 12:**
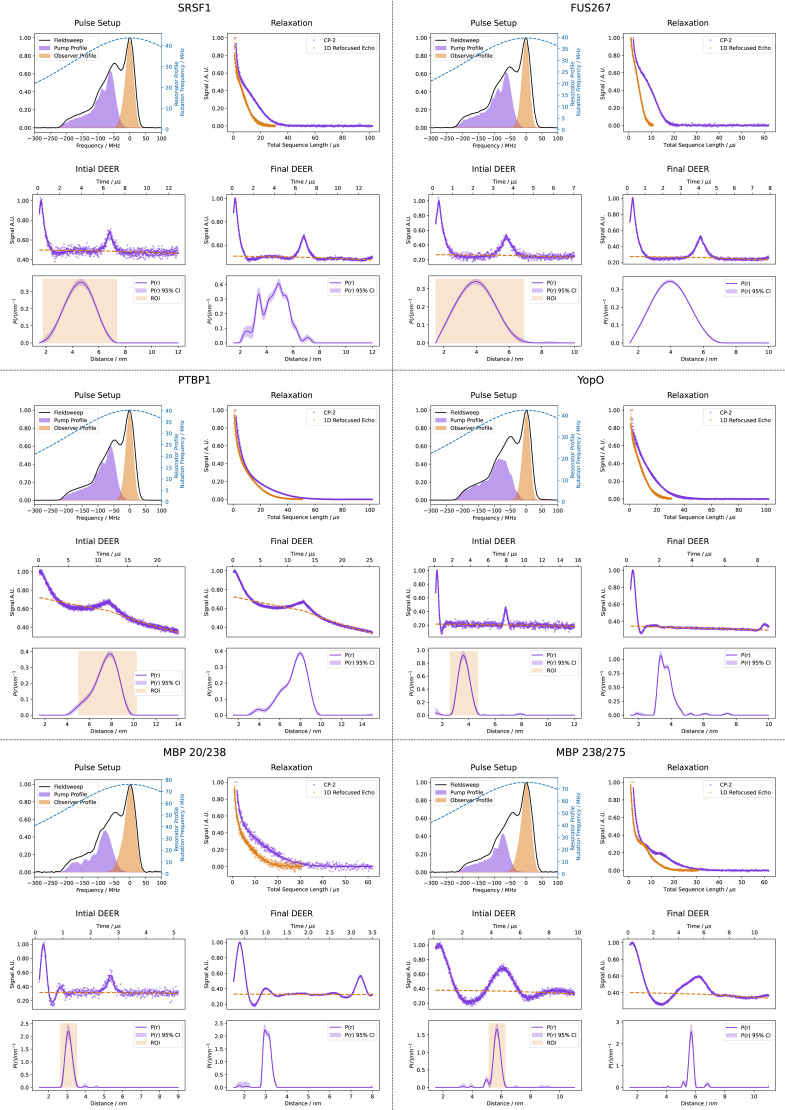
Results of the autoDEER protocol with samples 1–6, using the home-built AWG spectrometer. Plots within the panels represent key stages in the autoDEER protocol: excitation profiles of the pump and observer pulses with the EPR spectrum and the resonator profile; relaxation data; the initial DEER measurement with the time domain data at the top and the fitted distance distribution on the bottom as well as the region of interest (ROI) in light green; the final DEER measurement and the fitted distance distribution.

The performance of autoDEER is shown in Fig. 12. Each panel for each sample contains four plots. The first plot (top left) represents the pulse setup, showing both the observer sequence and the pump pulse excitation profile normalised to the echo-detected field sweep. Also shown is the fitted resonator profile. The second plot (top right) displays the relaxation traces for both the Carr–Purcell two-pulse decay and the 1D refocused-echo decay. The third plot (lower left) shows the initial DEER measurement, with the primary data in the top box and the distance distribution in the lower box. Additionally, the region of interest (ROI) is marked by a light-green box overlaying the distance distribution. The final plot (lower right) displays the final DEER measurement and the fitted distance distribution.

In all six cases, autoDEER was able to acquire high-quality DEER data in the allocated measurement time. The protocol achieved a highly optimized pulse setup for the specific spectrum and resonator profile. In the case of the YopO and MBP 20/238 samples it selected a short pump pulse to prevent broadening of the distance distribution. The protocol decided on a linear-chirped rectangular pump pulse in all cases. Different optimum observer pulse lengths were selected for the two resonators to account for the different *B*_1_ amplitudes. Detailed information about the respective pulse setup can be found in Table 1, and the pulse profiles are shown in the pulse setup figures in Fig. 12.

**Table 1:** Optimised pump and observer pulses for each measurement. The bandwidth of the rectangular pulses is estimated as 1/*t*_p_, where *t*_p_ is the pulse length, otherwise the bandwidth refers to the frequency sweep bandwidth

	Type	Length / ns	Bandwidth / MHz	Central Freq. / MHz
Sample 1: SRSF1				
Pump	Chirp	206.0	210.0	−141.6
Observer	Rect	12.0	83.3	−1.0

Sample 2: FUS267				
Pump	Chirp	206.0	220.0	−146.7
Observer	Rect	14.0	71.4	−1.3

Sample 3: PTBP1				
Pump	Chirp	206.0	220.0	−146.5
Observer	Rect	14.0	71.4	−1.0

Sample 4: YopO				
Pump	Chirp	72.0	220.0	−140.8
Observer	Rect	14.0	71.4	1.0

Sample 5: MBPL20S238				
Pump	Chirp	88.0	190.0	−137.6
Observer	Rect	8.0	125.0	2.6

Sample 6: MBPS238L275				
Pump	Chirp	206.0	200.0	−148.7
Observer	Rect	10.0	100.0	−0.3

In all cases, except for MBP 20/238 and YopO, autoDEER selected five-pulse DEER for the final measurement. The protocol switched to four-pulse DEER for the MBP 20/238 due to the shorter-pump pulse reducing the inversion efficiency, which has a greater impact on the dominant pathway for five-pulse DEER. The comparison of four-pulse *vs.* five-pulse DEER for all samples can be found in SI Fig. S9.

In all cases the initial DEER measurement correctly identified the region of interest (ROI) and gave a sufficiently good estimate of the modulation depth. In the YopO case, there is a drop in the modulation depth in the final DEER measurement. This is due to the selection of shorter pump pulses and their lower inversion efficiency at the available power. Note however that a high-quality DEER measurement was still obtained.

For all samples, the final DEER measurements achieved a high MNR and resulted in distance distributions with tight confidence intervals. The automated DeerLab DEER processing script was able to handle the different local concentrations and extract the non-modulated contribution (background). For MBP 238/275, large oscillations are noticed in the DEER trace, likely a consequence of the narrow distance distribution and of constructive interference between the main five-pulse pathway and the smaller four-pulse pathway. This did not cause any issues for the data processing.

### 5.1 Comparison to original data for the test samples

To determine whether our automated procedure is able to reproduce distance distributions the results from data acquired by experienced human operators, we compared the final DEER measurements with published data for the same samples. For consistency, we analysed the literature data with the same data processing procedure as used in autoDEER. In all cases the previously published measurements were performed with four-pulse DEER. Comparison of measurement times would not be reasonable as none of the published data was measured on the same experimental setup. Similarly, we do not compare modulation depths as the pulse setup is different, for the pulse setup of literature measurements see SI Table S10. All of the published data were measured using only monochromatic pulses, Gaussian pulses for the MBP samples and rectangular otherwise.

**Fig. 13:**
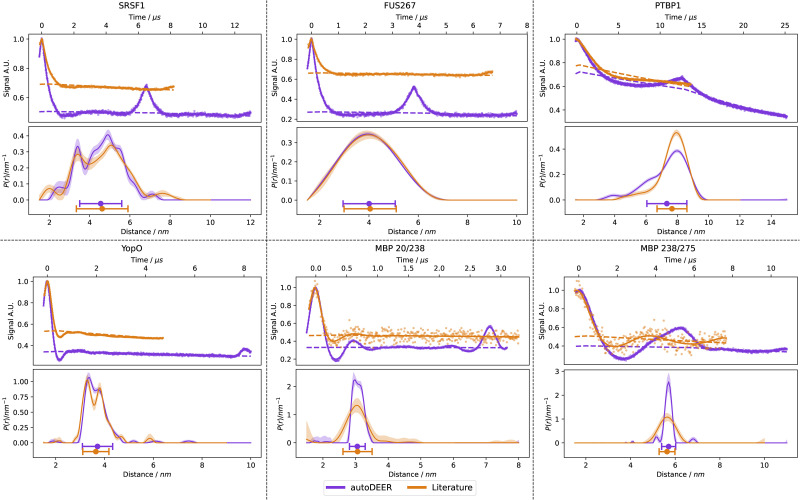
A comparison of the DEER traces of the six samples measured with the automated protocol (red) to the originally published results (teal). All the literature data was re-processed using the same procedure as for autoDEER, set out in SI Section S7. All literature measurements except for sample 2 (five-pulse DEER) were performed with the four-pulse DEER sequence. The mean and standard deviation of the distance distributions are compared below the distributions, with the dot and bar representing them, respectively.

Comparisons between the previously published data and the data obtained with automated application of our protocol are shown in Fig. 13. The means and standard deviations of the distance distributions are compared in Table 2. For all samples we find a good agreement with previously published data, with only small differences noticeable. In most cases, the automated procedure measured either a longer *τ*_evo_ or a higher MNR. The largest discrepancy in standard deviation is seen for MBP 238/275 and MBP 20/238 samples. This is likely due to the lower MNR in the literature data which led to a higher regularization parameter, which in turn broadened the distributions.

**Table 2:** Comparison of mean distances (*x̄*, nm) and standard deviations (*σ*, nm) between the test samples measured with autoDEER and previously published DEER data for the same samples. The 95% confidence intervals are shown in brackets

		autoDEER	Literature
SRSF1	*x̄*	4.55 (4.53, 4.57)	4.62 (4.60, 4.64)
	*σ*	1.04 (1.03, 1.06)	1.28 (1.26, 1.31)
FUS267	*x̄*	4.01 (4.00, 4.02)	4.05 (4.01, 4.08)
	*σ*	1.06 (1.05, 1.07)	1.06 (1.02, 1.10)
PTBP1	*x̄*	7.33 (7.29, 7.37)	7.66 (7.64, 7.68)
	*σ*	1.27 (1.21, 1.34)	0.95 (0.91, 1.00)
YopO	*x̄*	3.71 (3.71, 3.72)	3.64 (3.64, 3.65)
	*σ*	0.62 (0.60, 0.64)	0.54 (0.53, 0.55)
MBPL20S238	*x̄*	3.05 (3.04, 3.07)	3.06 (2.92, 3.20)
	*σ*	0.24 (0.09, 0.39)	0.45 (−0.11, 1.00)
MBPS238L275	*x̄*	5.71 (5.70, 5.71)	5.63 (5.51, 5.76)
	*σ*	0.31 (0.17, 0.46)	0.35 (−0.43, 1.13)

### 5.2 Bruker-AWG

**Fig. 14:**
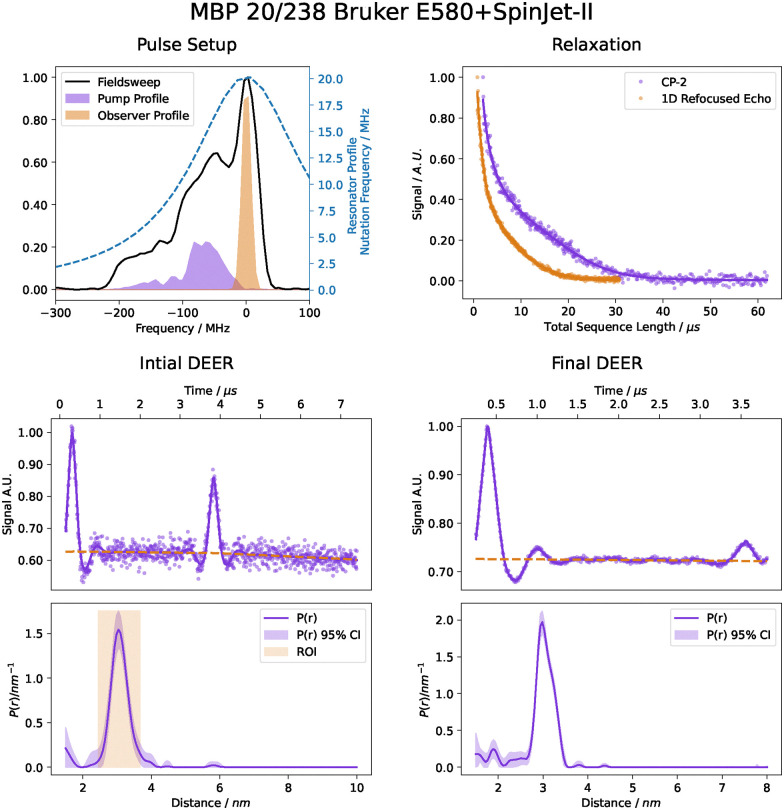
Results for the measurement of the MBP 20/238 sample on a Bruker ElexSys-II E580 spectrometer. Plots follow the same style as Fig. 12.

To demonstrate the applicability of this protocol on commercial hardware, we have implemented autoDEER on a Bruker ElexSys-II E580 spectrometer. The implementation is compatible with any Bruker ElexSys-II spectrometer which is equipped with either the SpinJet-I or SpinJet-II AWG add-on units and Xepr 2.9 software version. Fig. 14 shows the results of this test with the MBP 20/238 sample.

The data exhibit some differences to the data measured on our home-built spectrometer. Due to the lower power of the high-power amplifier and higher insertion losses in the bridge, there is considerably less *B*_1_ power than with the home-built spectrometer. The MBP 20/238 sample is particularly challenging with this low *B*_1_ power as it contains a short and narrow distance distribution so that only short chirp pulses are adequate. Hence, inversion performance is somewhat lower than with a higher-power amplifier. As in the case of the home-built spectrometer, autoDEER switched from an initial five-pulse DEER measurement to a final four-pulse DEER measurement because of limited power. Due to the more serious limitation on the commercial spectrometer, modulation depth is substantially smaller. Yet, data quality is still high and the distance distribution nicely matches the one obtained with the home-built spectrometer.

## 6 Conclusion and outlook

In this paper, we have set out a highly optimised fully-automated procedure for DEER experiments. Additionally, we have implemented this procedure in autoDEER, a free and open-source software package for conducting such experiments on both home-built and commercial spectrometers. We have also rigorously validated both autoDEER and the underlying protocol against a broad selection of published samples, ranging from simple rigid proteins with short distances to more complicated intrinsically disordered proteins. Both protonated and deuterated matrices were tested, showing the wide applicability of the protocol and of autoDEER.

The protocol presented does not assume the same measurement parameters for every sample but instead optimises parameters to the given sample conditions and experimental setup. The EPR spectrum and resonator profile are used to identify optimal pulses. Relaxation data are used to find optimal inter-pulse delays, such that the necessary MNR is reached in the available measurement time. An initial DEER measurement is used to estimate both the modulation depth and the distance range corresponding to the region of interest (ROI). These values are used to configure the final DEER measurement.

We have also developed a cross-platform user-friendly GUI that significantly simplifies the process of automation down to a single button whilst presenting publication quality plots at each stage of the protocol. This GUI allows non-experts to measure DEER with minimal introduction and without being exposed to the inherent complexities of an AWG-equipped pulse EPR spectrometer.

Currently, this protocol has been optimised specifically for nitroxide–nitroxide spin labelling; however, it is possible to expand it for use with other spin labels, such as copper and gadolinium labels, and to orthogonally spin-labelled samples in the future. The setup of an orthogonally labelled DEER experiment is often more complicated than for the nitroxide–nitroxide case. Hence, the use of automation could be even more beneficial in this case. Additionally, we do not consider orientation selection as for most nitroxide–nitroxide protein samples this is not significant.^12^ For rigid labels, where orientation selection is noticeable, operators would need to follow a more advanced protocol.^54^

The main limitations of this protocol stem from the fact that most pulse EPR spectrometers are not designed for automation. One such limitation is that the resonator frequency must be roughly known before starting, as very few pulse EPR spectrometers have a high-quality tune mode where the resonator mode can be quickly identified purely from power reflection. Another limitation is that resonators do not have a digitally controllable coupling, which would allow for the automation and optimisation of the resonator coupling. Other current hardware limitations include limited AWG memory and lack of support for non-uniform sampling. In both cases the necessary technology to circumvent these issues exists, but is not yet implemented in established spectrometer designs.

Currently, very few spectrometers, either commercial or home-built, have been designed for automated spectroscopy. As new spectrometer models are released, automated EPR spectroscopy in DEER and beyond will become more widely available. Nonetheless, we have demonstrated that an automated protocol can be implemented on current commercial spectrometers. This opens up automated DEER spectroscopy to a wide-range of laboratories worldwide with only limited additional investment required.

The code base for autoDEER is available online open-source on GitHub, ensuring that the code and the underlying protocol are fully transparent, enabling community-driven development and adaptation to the latest developments. As both DEER as a technique and EPR instrumentation progress, it is important that the protocol is also further developed.

By simplifying and optimising the measurement process, automated DEER spectroscopy has the potential to make DEER accessible to researchers focusing on other techniques in molecular and structural biology, in particular to researchers that wish to complement bio-NMR by EPR. This process could be further enhanced by the development of spectrometer hardware that is specifically designed for automation. Finally, automation has the potential to be applied in pulse EPR beyond DEER spectroscopy, for instance in hyperfine spectroscopy, enabling the design of more sophisticated experiments, expansion of the reach of such measurements beyond method-developing EPR groups, and an increase of the experimental throughput with given spectrometer capacity.

## Author contributions

Conceptualization: HK, GJ; methodology: HK, SK, SS, GJ; investigation: HK; formal analysis: HK; visualization: HK; software: HK; supervision: SS, GJ; writing – original draft: HK; writing – review and editing: HK, SK, SS, GJ.

## Conflicts of interest

There are no conflicts to declare.

## Supplementary Material

CP-028-D5CP03536H-s001

## Data Availability

The data supporting this article have been included as part of the supplementary information (SI). Supplementary information: all of the scripts for generating the figures and data analysis have been included and are available under the MIT license. See DOI: https://doi.org/10.1039/d5cp03536h. Data for this article, including Jupyter data analysis notebooks and experimental data are available at Zenodo at https://doi.org/10.5281/zenodo.17543328. The code for autoDEER can be found at https://github.com/JeschkeLab/autoDEER with DOI https://doi.org/10.5281/zenodo.17107364. The version of the autoDEER code employed for this study is version 1.0. The code for PyEPR, which is the backend for autoDEER, can be found at https://github.com/JeschkeLab/PyEPR with DOI: 10.5281/zenodo.17107011. The version of the PyEPR code employed for this study is version 1.0. Both autoDEER and PyEPR have been published under the GNU General Purpose License version 3.
