# Supplementary material for: Towards full optimisation of automated double electron–electron resonance spectroscopy
Source: Phys Chem Chem Phys. 2025 Dec 8;28(2):1241–59. doi: 10.1039/d5cp03536h (PMC12699958; doi:10.1039/d5cp03536h)
Supplement: CP-028-D5CP03536H-s001 [file CP-028-D5CP03536H-s001.pdf]

Supplementary Information (SI) for Physical Chemistry Chemical Physics.
This journal is © the Owner Societies 2025

Towards Full Optimisation of Automated Double Electron–Electron Resonance Spectroscopy

Hugo Karas, Sergei Kuzin, Stefan Stoll, Gunnar Jeschke*

Contents

S1 Resonator Profile **2**

S2 DEER pulses Optimisation **3**
S2.1 Pulse Excitation Profile Calculation . . . 3
S2.2 Optimal DEER pulse setup for different pulse types . . . 4

S3 2D Refocused Echo Scans **5**

S4 Carr-Purcell Relaxation **6**

S5 Optimal DEER delays **7**
S5.1 Simulated distributions . . . 7
S5.2 t_{min} . . . 8
S5.3 Δt . . . 8

S6 Optimal DEER MNR **9**

S7 Automated DEER fitting procedure **10**

S8 SRT scan comparison **12**

S9 Optimal τ_{evo} for four- and five-pulse DEER **13**

S10 Literature Measurement Pulse Setup **14**

S1 Resonator Profile

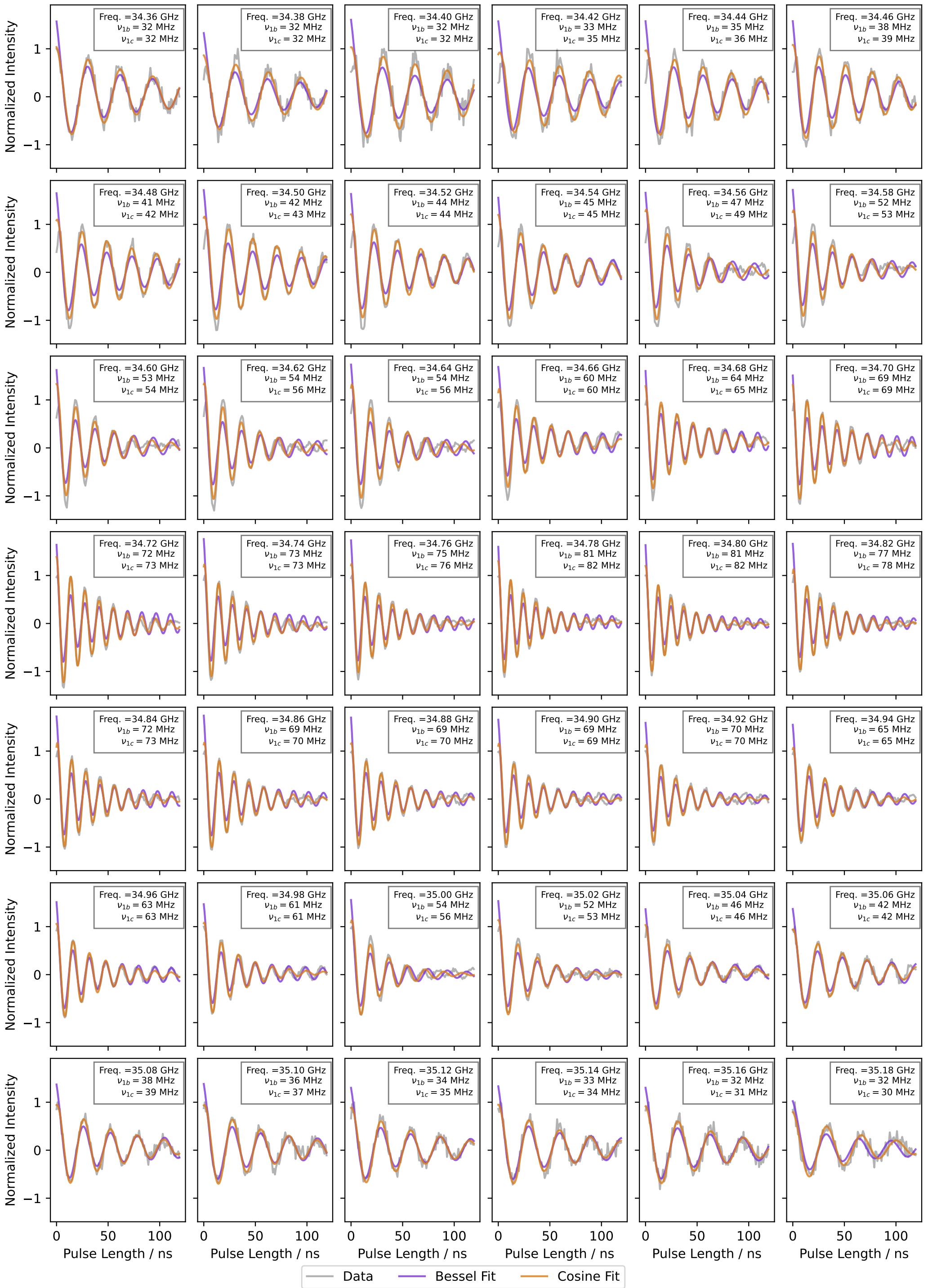

Figure S1: A comparison of the fitting of a resonator profile experiment between a cosine fit (orange) and a Bessel fit (purple colour). The sample was MBP L20/S238 measured in the 1.6 mm Pent Loop Gap resonator on the homebuilt ETH spectrometer. The original data can be seen in fig. 3 of the main text.

S2 DEER pulses Optimisation

S2.1 Pulse Excitation Profile Calculation

The pulse inversion simulations were done using the two-level approximation simulation model [1]. In this model, we piecewise investigate how the pulse excites transitions between two energy levels, assuming the system is $S = 1/2$, and consider the time evolution under the time-dependent Schrödinger equation.

The Hamiltonian $\hat{\mathcal{H}}(t)$ is approximated as

$$\hat{\mathcal{H}}(t) = \Delta\omega \hat{S}_z + \omega_1(t)[\hat{S}_x I(t) + \hat{S}_y Q(t)] \quad , \tag{1}$$

where $\Delta\omega$ is the frequency offset, I, Q are the in-phase and quadrature components of the B_1 field, i.e. the pulse.

This is solved piecewise, by defining a propagator $\hat{U}_k$ for a small-time step Δt and assuming that Hamiltonian is constant with the initial magnetisation being $\hat{M}_0 = (0, 0, 1)$

$$\hat{U}_k = \exp(-i\hat{\mathcal{H}}_k \Delta t) \quad , \tag{2}$$

where the result $\hat{U}$ is determined by the product

$$\hat{U} = \prod_{i=1}^{N} \hat{U}_{N-i} \quad . \tag{3}$$

The M_z component can then be extracted from $\hat{U}$ as $\mathrm{tr}(\hat{S}_z \hat{U} \hat{S}_z \hat{U}^\dagger)/\mathrm{tr}(\hat{S}_z^2)$.

To determine the excitation profile of a π pulse the M_z component is then re-normalised by

$$P_\pi = \frac{-M_z + 1}{2} \quad . \tag{4}$$

For a $\pi/2$ pulse we are interested in the total transverse relaxation excitation profile.

$$P_{\pi/2} = \sqrt{M_x^2 + M_y^2} \equiv \sqrt{1 - M_z^2} \quad . \tag{5}$$

The pulse train excitation profiles, P_{obs} and P_{pump}, are calculated as a product of all the pulses in the sequence.

$$P_{\mathrm{obs}} = P_{\mathrm{obs},\pi/2} \cdot P_{\mathrm{obs},\pi}^2 \tag{6}$$

$$P_{\mathrm{pump}} = P_{\mathrm{pump},\pi}^N \tag{7}$$

where, N is the number of pump pulses.

Since we need the probability profiles $\bar{P}$ that the spin is exclusively observed or pumped, we need to subtract the overlap.

$$\bar{P}_{\mathrm{obs}} = P_{\mathrm{obs}} - P_{\mathrm{obs}} P_{\mathrm{pump}} \tag{8}$$

$$\bar{P}_{\mathrm{pump}} = P_{\mathrm{pump}} - P_{\mathrm{obs}} P_{\mathrm{pump}} \tag{9}$$

S2.2 Optimal DEER pulse setup for different pulse types

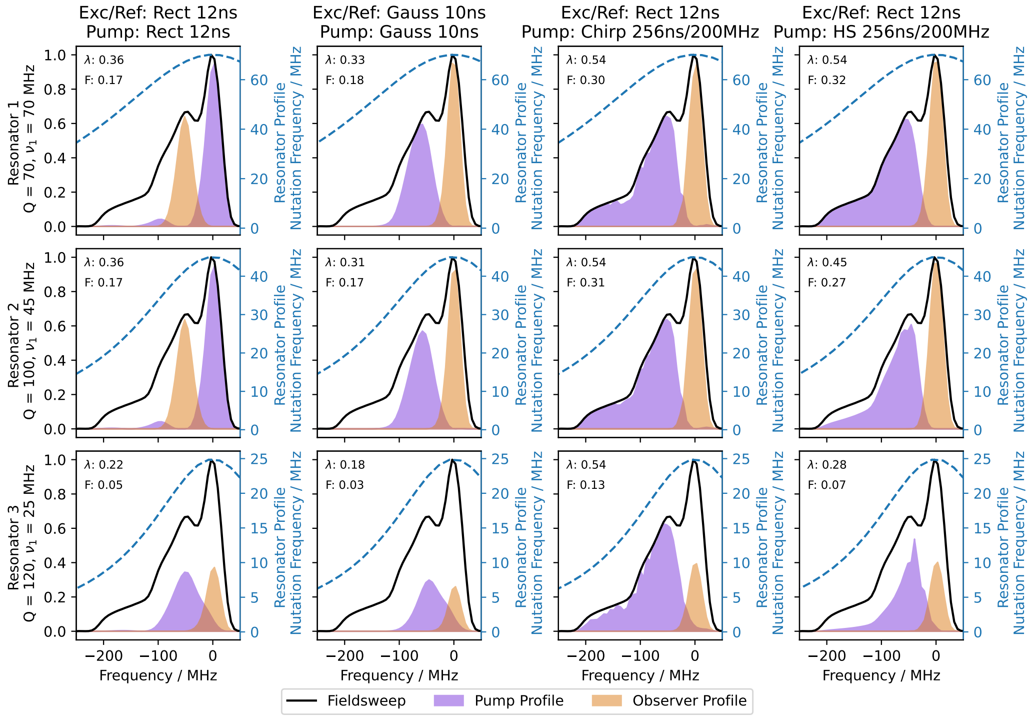

Figure S2: A comparison of the optimal pulse setup for different pulse pairings (X-axis) and different resonators (Y-axis). The modulation depth is given by λ and the sensitivity by F.

S3 2D Refocused Echo Scans

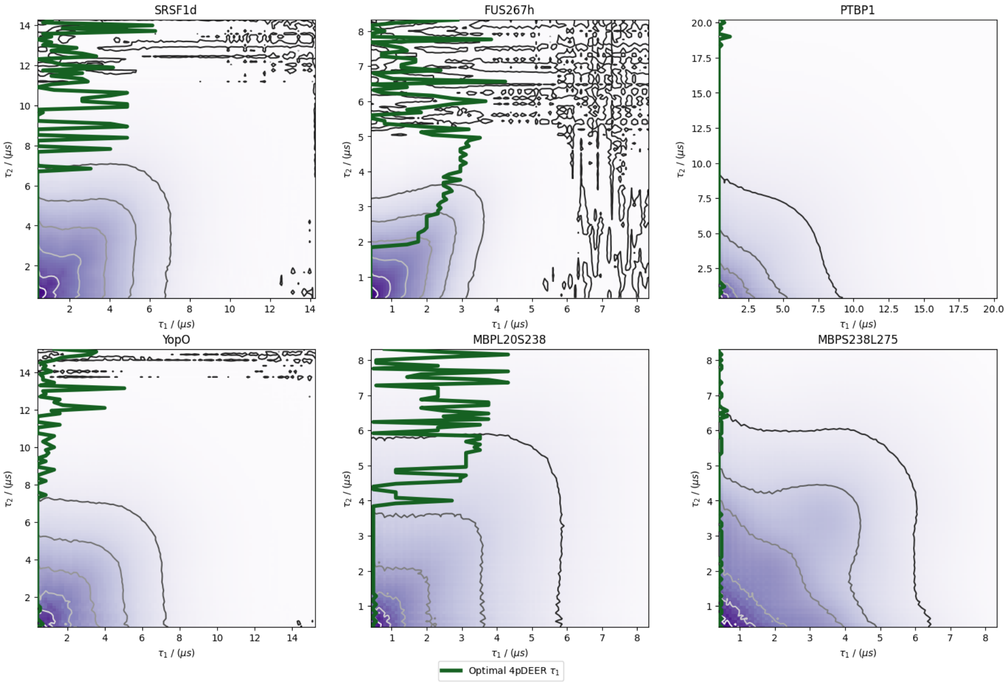

Figure S3: A comparison of the 2D refocused echo decay is shown for all the samples in our sample set. The optimal τ_1 for each τ_2 for a four-pulse DEER measurements is shown with the red dashed line, where the surface is essentially flat with respect to the noise level there is a large variation in the optimal τ_1, however the difference is minimal. Only the FUS267h sample has a fully protonated matrix and it is here that the use of a longer τ_1 is most noticeable.

S4 Carr-Purcell Relaxation

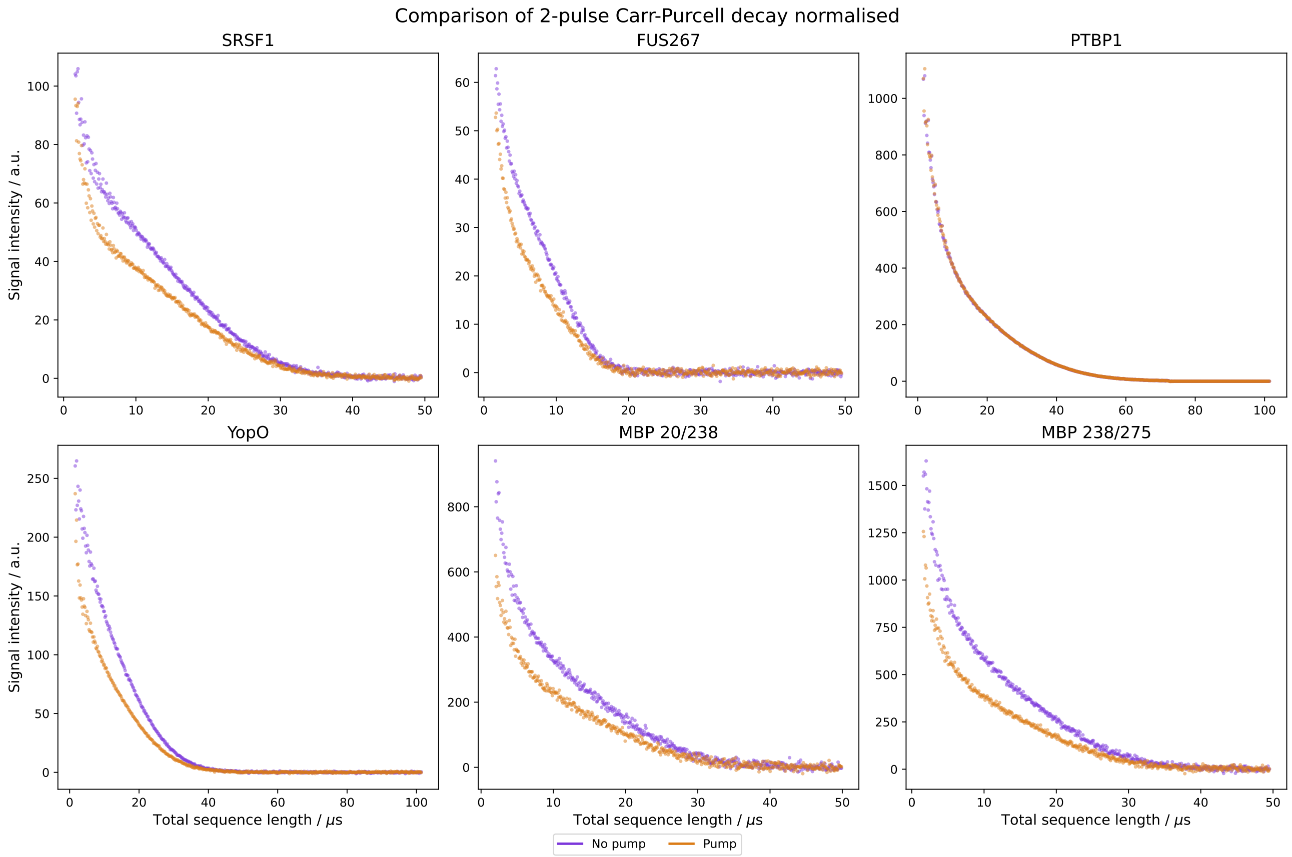

Figure S4: A comparison of the 2-pulse Carr-Purcell decay is shown, with and without the pump pulse present for all samples in our data set. The x-axis is the total sequence length (equals to 4τ). The data without the pump pulse is shown in purple and the data with it is shown in orange. The measurements have been normalised by number of acquisitions to show differences in signal intensity. All measurements were done with 12 ns rectangular observer pulses at the spectral maximum and a 12 ns pump pulse 80 MHz lower in frequency.

S5 Optimal DEER delays

Due to the ill-posed nature of the inverse problem that is extracting a distance distribution from a DEER trace, it can be very challenging to extract the consequence of adjusting parameter selection. For this reason we will conduct a Monte Carlo based simulation approach to determine the effect of different DEER parameters on the quality of the DEER trace.

We have generated a set of three different distance distributions, and from each of them nine different time domain traces. The time domain traces are generated by DeerLab using the *dipolarmodel* function. We will then add Gaussian-white noise to these time domain traces to simulate experimental conditions, this step will be repeated 40 times with different seed values to generate different noise distributions. The noise is added such that the MNR is equal to 50, unless otherwise stated.

To compare the fitted distributions v, to the ground truth input u, we use the statistical Wasserstein distance, also known as the earth movers distance (EMD) and defined as,

$$W(u(r), v(r)) = \int |U - V| \mathrm{d}r \quad , \tag{10}$$

where U and V are the respective cumulative density function of the distributions u and v.

S5.1 Simulated distributions

Only short rigid distances were simulated as there are the situations where under sampling or incorrect identification of the refocusing time will be most noticeable. For simplicity most distributions are Gaussian in shapes, except for distribution 1 which is bi-Gaussian. From each distributions, three τ_{evo} lengths were calculated and for each of these three different concentrations were simulated with a homogenous 3D background. This gives a total of nine different DEER traces for each distribution.

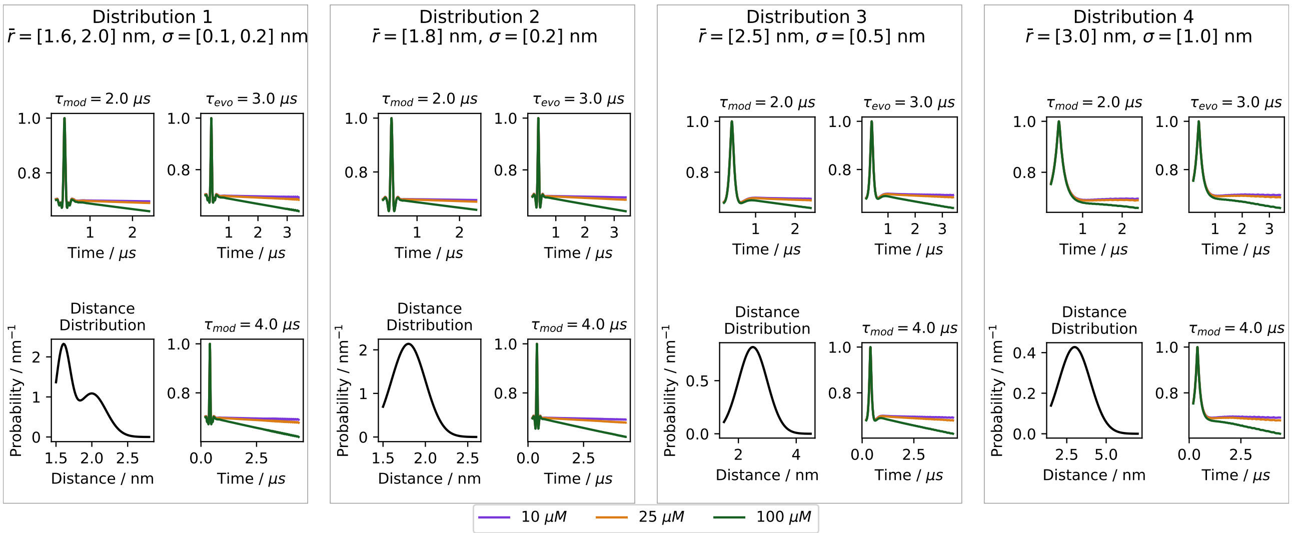

Figure S5a: A figure showing the four distributions used for determining the optimal DEER parameters. In each sub-figure there, the bottom left shows the distribution and the others in clockwise order show $\tau_{\mathrm{evo}} = 2,\ 3,\ 4\ \mu s$, where three concentrations are simulated 10, 25 and 100 μM.

S5.2 t_{min}

In this investigation we define t_{min} with respect to the dominate pathway, i.e. for $\tau_1 = 400$ ns and $t_{\mathrm{min}} = -150$ ns, the first point if at 250 ns. In Fig. S5b, we can see that changing the t_{min} only has a mild effect on the distance distribution as long as $t_{\mathrm{min}} < -0.050$ μs. We have chosen to use a value of $t_{\mathrm{min}} = -0.150$ μs.

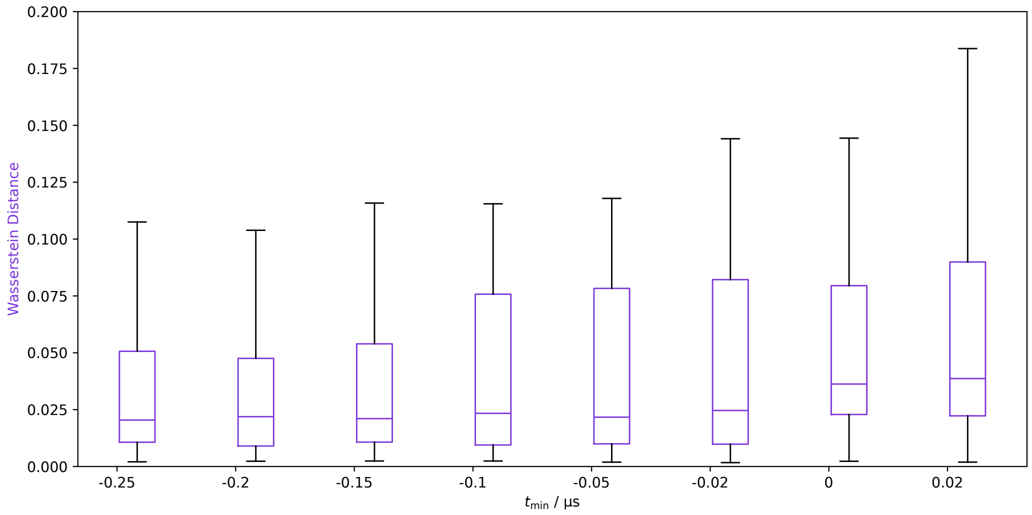

Figure S5b: A box-plot of the Wasserstein Distance comparing the fitted distribution to the ground truth for different t_{min}

S5.3 Δt

The given MNR value is for $\Delta t = 16$ ns, MNR is adjusted for larger and smaller Δt to preserve equivalent measurement time. Only the first two distributions were used in the simulation as they will have the greatest impact from undersampling.

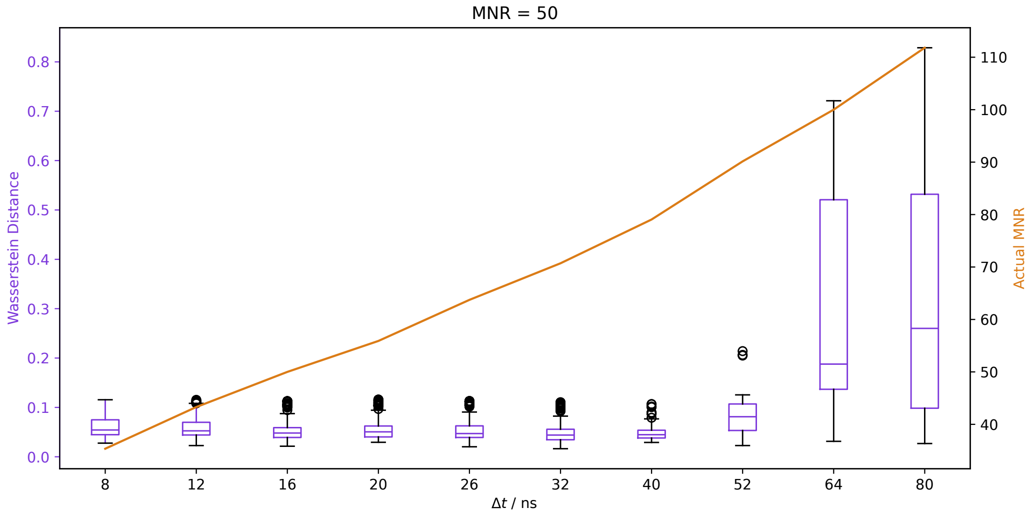

Figure S5c: A box-plot of the Wasserstein Distance comparing the fitted distribution to the ground truth for different Δt in purple. The corrected MNR is given in orange.

S6 Optimal DEER MNR

We reuse the distributions shown in Fig. S5a to simulate the effect of different MNR on the fitted distributions. This is shown in Fig. S6a. In this case we have also investigated how the mean and standard deviation of the distribution changes.

In Fig. S6b, we investigate how the ROI calculated using a cumulative integral method with a 95% coverage depends on the MNR. The percentage error is shown. Since all distance distributions simulated have low distances that are non-zero at the sensitivity limit of $r = 1.5$ nm there is little noticeable, this effect is exaggerated as the distances are small. The upper limit however shows improvements with greater MNR, however by an MNR of 20 there is less than a 5% error.

We can conclude from these figures for determining the rough mean and standard deviation a MNR of 20 is sufficient, however to determine the shape of the distribution a MNR > 100 is required. A much larger MNR > 250 serves no advantage.

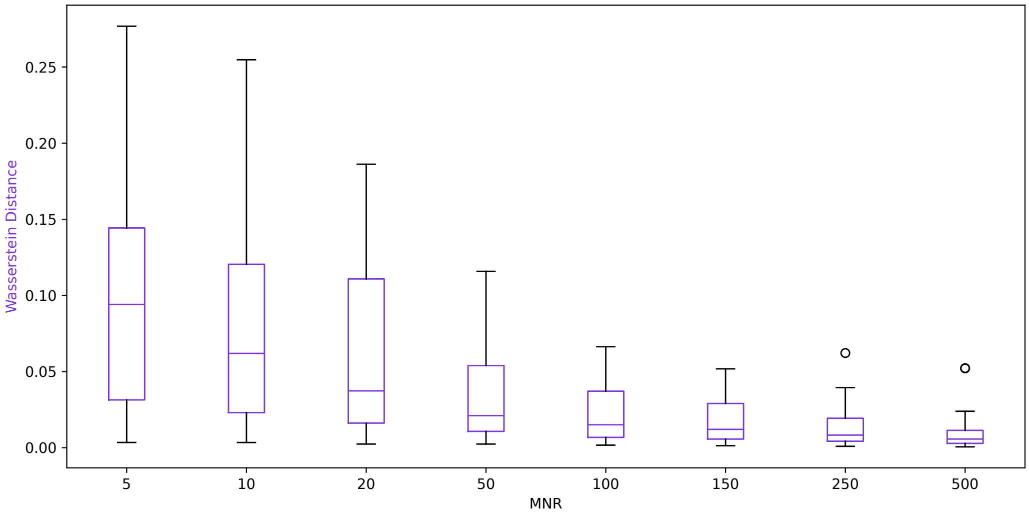

Figure S6a: A box-plot of the Wasserstein Distance comparing the fitted distribution to the ground truth for different MNR in pink.

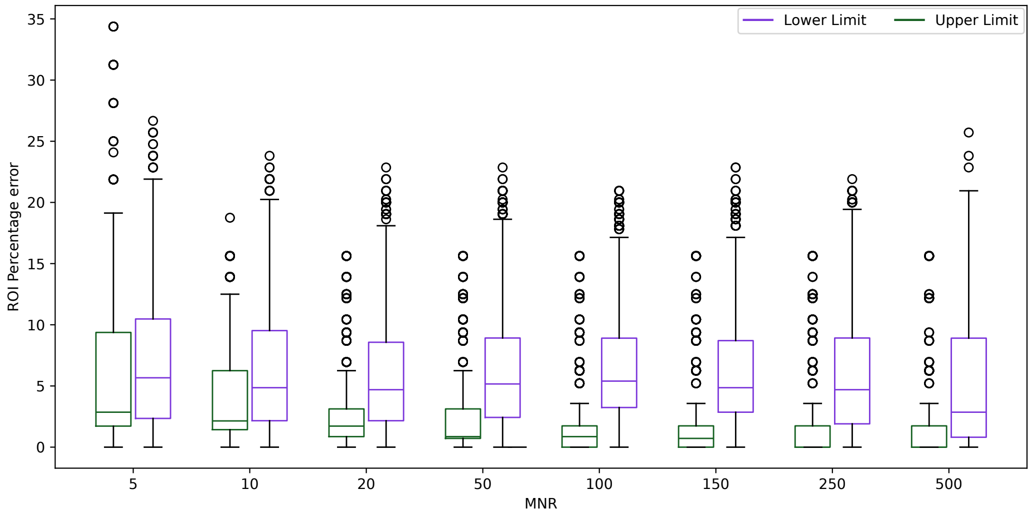

Figure S6b: A box-plot of the percentage error, with respect to the ground truth, in the upper (green) and lower (purple) bound of the distance distribution ROI for different MNR.

S7 Automated DEER fitting procedure

A crucial component of this protocol is the automated analysis of the data, both during and after their acquisition. These two situations differ in their requirements. During the experiment, the MNR is often low and the processing needs to be reasonably fast (less than 5 minutes). After the experiment is complete, the focus is on high quality of the processing, which can take longer.

All data analysis is performed using the latest version of DeerLab (v1.1.4) [2]. We chose DeerLab, first, because its Python-based architecture is easy to integrate into our software package. Second, DeerLab can handle multiple pathways which is essential for processing five-pulse DEER data and may be beneficial for four-pulse DEER data which often feature contributions from a "2+1" pathway.[3] Third, DeerLab implements the *compactness criterion* which adds an additional constraint and significantly improves the identifiability of background and subsequently the extraction of a high-quality distance distribution [4].

Step 1: Preparing the inputs The dataset saved by the PyEPR package automatically saves the relevant metadata. This metadata contains (among other information):

- the inter-pulse delays (τ_1, τ_2...)
- the time axis (as defined in Fig. 10)
- the phase cycle program.

When using a non-parametric distance distribution model, there has long been a dependence on having a correctly defined r axis. The r axis should be as tight as possible whilst not excluding any distances. Although the compactness criterion reduces sensitivity of the fit to the choice of the distance axis, it may still fail for an unreasonable choice. Therefore, we set the r axis using

$$1.5 \text{ nm} \leq r \leq \left\lceil \sqrt[3]{\frac{\tau_{\text{evo}} \cdot 6^3}{2}} \right\rceil \text{ nm} \tag{11}$$

where the upper bound represents the maximal distance at which a mean distance can be reliably determined [5], and $\lceil\rceil$ symbol denotes the integer ceiling function. DeerLab's compactness criterion is sufficiently stable to a longer r axis and to allows for integer rounding to be used.

Additionally, robustness of the fit depends on the number of points. Too few points will hinder the identification of the optimal regularisation parameter and result in a less smooth distribution. Too many points will increase the number of parameters and the fit time. Normally between 100 and 300 points is a good compromise. We set the number of points to 150, except when becomes $\Delta r < 0.1\,\text{nm}$ then we increase to 300 points.

Step 2: Identifying crossing echoes (if applicable) Crossing echoes may appear in DEER traces, depending on the phase cycling method used [6]. This is particularly prevalent in five-pulse DEER measurements conducted with an incoherent ELDOR source and only a 2-step DC offset removal phase cycle. This step is only done if a crossing echo is expected, otherwise it is skipped. These crossing echoes can be identified through the out-of-phase component of the phase-corrected signal, as they maintain an inconsistent phase relative to the primary signal and thus remain unaffected by phase correction.

To mitigate the impact of crossing echoes, we implement a data mask in the DeerLab fitting process[7]. This mask is generated by selecting data points that meet two criteria: Firstly, they exceed the noise threshold, defined as $\text{Im}(V_i) > 4\sigma$, where V_i represents a single data point and σ denotes the standard deviation of the DEER trace (V), and secondly, they fall within $\pm tp_{\max}$ of an expected crossing echo, where $tp_{\max}$ is the maximum pulse length. To ensure comprehensive removal, we also exclude three adjacent data points on either side of each identified crossing echo.

Step 3: Optimising the number of pathways In multiple-pathway DEER analysis, it is important to avoid over-fitting due to a too complex model while at the same time including all relevant contributions. Typically, DEER experiments exhibit one or two (in the case of five-pulse) dominant pathways, with additional pathways contributing significantly smaller amplitudes. These minor pathways will have an MNR smaller than that of the main pathway(s) and should only be included in the analysis if their signal exceeds the noise level of the DEER trace.

To minimise the number of parameters in the fitting process, we can use prior knowledge about the DEER experiment. For instance, certain pathways may have equivalent dipolar characteristics, resulting in identical probability and modulation depth parameters. By identifying and consolidating these equivalent pathways, we further streamline the model and improve the robustness of the fit.

Step 4: Configuring the model Multi-pathway DEER analysis significantly increases the number of parameters, especially if each parameter is modelled with its own free-floating refocusing time. DeerLab enables the reparametrisation of the model from refocusing times into inter-pulse delays ($\tau_1,\ \tau_2,\ \ldots$), which would reduce the number of time parameters from 4 to 2 in four-pulse DEER and from 5 to 3 in five-pulse DEER, assuming that all pathways are modeled. This reduced number of parameters not only reduces the fitting time but also increases the stability of the fit.

The lower and upper bounds of fitted time parameters, either refocusing time or inter-pulse delays, are set in DeerLab from the pulse length. When chirp pump pulses are used the refocusing time is often shifted and thus these bounds need to be extended. However, if they are too large the fitting algorithm is given too much freedom and can converge incorrectly. We recommend allowing for a minimum of ±40 ns.

By default, in DeerLab, the lower and upper bounds of the modulation depths are set to 0 and 1 respectively. This flexibility is unrealistic in most DEER situations and certainly the ones generated by this protocol, and thus we suggest that all minor pathways occurring from the incomplete flipping of the refocusing pulses are set to an upper bound of 0.2. These pathways are denoted as pathways [2,3,4] in DeerLab. Restricting these bounds reduces the risk that a local minimum is optimised, with non-physical modulation depths especially in samples with a rigid distance.

Step 5: Background model We assume a homogenous 3D background model [7, 8]. However, the operator can set a different background model for cases such as membrane proteins. The homogenous 3D background function is given by

$$B(t) = \exp\left(-\frac{8\pi^2}{9\sqrt{3}}\lambda_0 c_\mathrm{s} D|t|\right), \tag{12}$$

where λ_0 is the inversion efficiency of the pump pulse and c_s is the total spin concentration. A difference between λ_0 and the modulation depth λ arises for incomplete spin labelling, which is very often encountered. In this case, $\lambda = f\lambda_0$, where f is an effective spin labelling efficiency. By using the resonator profile, EPR spectrum and the pulse profiles, we estimate λ_0 using eq. 12. Thus, total spin concentration c_s can be obtained from the background fit.

Step 6: Configuring the fitting DeerLab offers a variety of fitting options that influence both the quality and efficiency of the analysis. Our aim is to balance computational efficiency and accuracy in our fitting. As a compromise between quality and computational effort, we use the following standard configuration:

1. **Solver selection:** The non-negative least squares (nnls) solver is set to use the 'quadprog' package, which implements the Goldfarb/Idnani dual algorithm [9]. This solver has been shown to produce superior fits in significantly less time than the default solver, although its availability may be limited on some systems.

2. **Regularisation parameter:** The choice of selection criteria for the regularisation parameter can significantly impact the fitted distance distribution [7, 10]. Based on our extensive testing, we have found that the *Bayesian Information Criterion* (BIC) or *Akaike Information Criterion* (AIC) consistently yield the most reliable results [11].

Experienced operators can override the selection criterion for the regularisation parameter or provide a preset regularization parameter for a series of samples where distance distributions need to be compared.

S8 SRT scan comparison

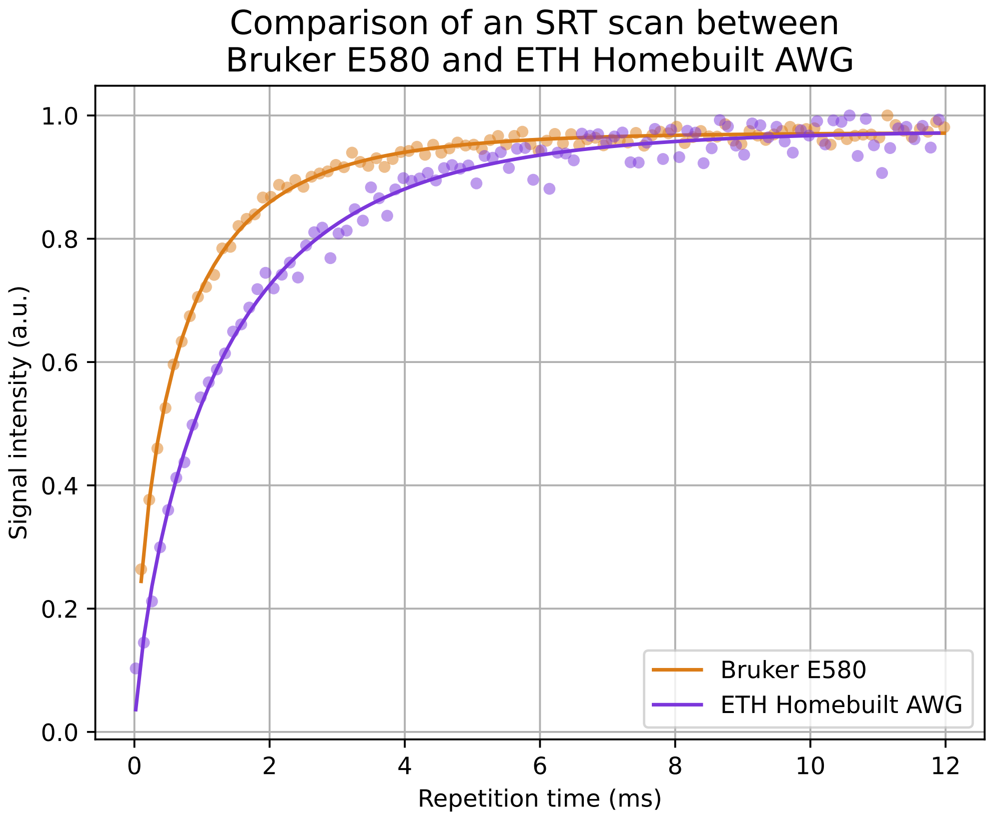

Figure S8: A comparison of the shot repetition time scan of the MBP L20/S238 sample on our homebuilt AWG spectrometer (purple) and a Bruker E580 (orange)

S9 Optimal τ_{evo} for four- and five-pulse DEER

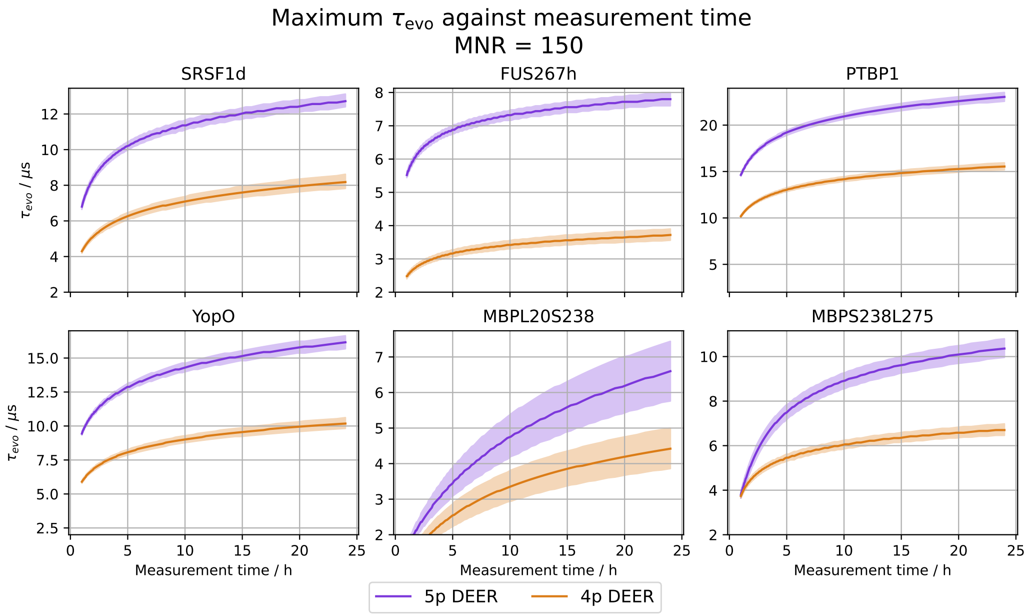

Figure S9: For the test measurements of the sample set on the ETH homebuilt spectrometer the comparison in τ_{evo} for $MNR = 150$ between four- (orange) and five- (purple) pulse DEER.

S10 Literature Measurement Pulse Setup

Sample 1: SRSF1d [12]				
	Type	Length ns	Bandwidth MHz	Central Freq. MHz
Pump	Rect	16.0	62.5	-0.0
Observer	Rect	16.0	62.5	-100.0
**Sample 2: FUS267h [13]**				
	Type	Length ns	Bandwidth MHz	Central Freq. MHz
Pump	Rect	16.0	62.5	0.0
Observer	Rect	16.0	62.5	-100.0
**Sample 3: PTBP1 [14]**				
	Type	Length ns	Bandwidth MHz	Central Freq. MHz
Pump	Rect	12.0	83.3	0.0
Observer	Rect	12.0	83.3	-100.0
**Sample 4: YopO [15]**				
	Type	Length ns	Bandwidth MHz	Central Freq. MHz
Pump	Rect	12.0	83.3	0.0
Observer	Rect	12.0	83.3	-100.0
**Sample 5: MBPL20S238 [16]**				
	Type	Length ns	Bandwidth MHz	Central Freq. MHz
Pump	Gauss	60.0	51.0	0
Observer	HS	150.0	80	-80
**Sample 6: MBPS238L275 [16]**				
	Type	Length ns	Bandwidth MHz	Central Freq. MHz
Pump	Gauss	60.0	51.0	0.0
Observer	HS	150.0	80.0	-80.0

Table S10: Optimised pump and observer pulses for each measurement. The bandwidth of the rectangular pulses is estimated as $1/t_\mathrm{p}$, and for a Gaussian pulse $2\sqrt{2\ln 2}/t_\mathrm{p}$[17], where t_p is the pulse length, otherwise the bandwidth refers to the frequency sweep bandwidth. 'Gauss' refers to a Gaussian pulse and 'HS' refers to a sech/tanh "hyperbolic secant" pulse with $\beta = 10$.

References

(1) G. Jeschke, S. Pribitzer and A. Doll, *The Journal of Physical Chemistry B*, 2015, **119**, 13570–13582.

(2) L. Fábregas Ibáñez, G. Jeschke and S. Stoll, *Magnetic Resonance*, 2020, **1**, Publisher: Copernicus GmbH, 209–224.

(3) L. Fábregas-Ibáñez, M. H. Tessmer, G. Jeschke and S. Stoll, *Physical Chemistry Chemical Physics*, 2022, **24**, 2504–2520.

(4) L. Fábregas-Ibáñez, G. Jeschke and S. Stoll, *Journal of Magnetic Resonance*, 2022, **339**, 107218.

(5) G. Jeschke, in *EPR Spectroscopy: Fundamentals and Methods*, Wiley, 2018.

(6) C. E. Tait and S. Stoll, *Physical Chemistry Chemical Physics*, 2016, **18**, 18470–18485.

(7) L. Fábregas Ibáñez, Ph.D. Thesis, ETH Zurich, 2022.

(8) A. D. Milov, A. B. Ponomarev and Y. D. Tsvetkov, *Chemical Physics Letters*, 1984, **110**, 67–72.

(9) D. Goldfarb and A. Idnani, *Mathematical Programming*, 1983, **27**, 1–33.

(10) L. Fábregas Ibáñez and G. Jeschke, *Journal of Magnetic Resonance*, 2019, **300**, 28–40.

(11) T. H. Edwards and S. Stoll, *Journal of Magnetic Resonance*, 2018, **288**, 58–68.

(12) Laura Esteban Hofer, Ph.D. Thesis, ETH Zurich, 2022.

(13) L. Esteban-Hofer, L. Emmanouilidis, M. Yulikov, F. H.-T. Allain and G. Jeschke, *Biophysical Journal*, 2024, **123**, 538–554.

(14) G. Dorn, C. Gmeiner, T. De Vries, E. Dedic, M. Novakovic, F. F. Damberger, C. Maris, E. Finol, C. P. Sarnowski, J. Kohlbrecher, T. J. Welsh, S. Bolisetty, R. Mezzenga, R. Aebersold, A. Leitner, M. Yulikov, G. Jeschke and F. H.-T. Allain, *Nature Communications*, 2023, **14**, 6429.

(15) O. Schiemann, C. A. Heubach, D. Abdullin, K. Ackermann, M. Azarkh, E. G. Bagryanskaya, M. Drescher, B. Endeward, J. H. Freed, L. Galazzo, D. Goldfarb, T. Hett, L. Esteban Hofer, L. Fábregas Ibáñez, E. J. Hustedt, S. Kucher, I. Kuprov, J. E. Lovett, A. Meyer, S. Ruthstein, S. Saxena, S. Stoll, C. R. Timmel, M. Di Valentin, H. S. Mchaourab, T. F. Prisner, B. E. Bode, E. Bordignon, M. Bennati and G. Jeschke, *Journal of the American Chemical Society*, 2021, **143**, 17875–17890.

(16) M. H. Tessmer, E. R. Canarie and S. Stoll, *Biophysical Journal*, 2022, **121**, 3508–3519.

(17) M. Teucher and E. Bordignon, *Journal of Magnetic Resonance*, 2018, **296**, 103–111.
